# The Stress-Strain Data of the Hip Capsule Ligaments Are Gender and Side Independent Suggesting a Smaller Contribution to Passive Stiffness

**DOI:** 10.1371/journal.pone.0163306

**Published:** 2016-09-29

**Authors:** Philipp Pieroh, Sebastian Schneider, Uwe Lingslebe, Freddy Sichting, Thomas Wolfskämpf, Christoph Josten, Jörg Böhme, Niels Hammer, Hanno Steinke

**Affiliations:** 1 Department of Orthopedics, Trauma and Plastic Surgery, University of Leipzig, Leipzig, Germany; 2 Department of Anatomy and Cell Biology, Martin Luther University Halle-Wittenberg, Halle (Saale), Germany; 3 Institute of Sport Science, Department of Human Locomotion, Chemnitz University of Technology, Chemnitz, Germany; 4 Institute of Anatomy, University of Leipzig, Leipzig, Germany; 5 Department of Anatomy, University of Otago, Dunedin, New Zealand; University of Zaragoza, SPAIN

## Abstract

**Background:**

The ligaments in coherence with the capsule of the hip joint are known to contribute to hip stability. Nevertheless, the contribution of the mechanical properties of the ligaments and gender- or side-specific differences are still not completely clear. To date, comparisons of the hip capsule ligaments to other tissues stabilizing the pelvis and hip joint, e.g. the iliotibial tract, were not performed.

**Materials & Methods:**

Hip capsule ligaments were obtained from 17 human cadavers (9 females, 7 males, 13 left and 8 right sides, mean age 83.65 ± 10.54 years). 18 iliofemoral, 9 ischiofemoral and 17 pubofemoral ligaments were prepared. Uniaxial stress-strain properties were obtained from the load-deformation curves before the secant elastic modulus was computed. Strain, elastic modulus and cross sections were compared.

**Results:**

Strain and elastic modulus revealed no significant differences between the iliofemoral (strain 129.8 ± 11.1%, elastic modulus 48.8 ± 21.4 N/mm^2^), ischiofemoral (strain 128.7 ± 13.7%, elastic modulus 37.5 ± 20.4 N/mm^2^) and pubofemoral (strain 133.2 ± 23.7%, elastic modulus 49.0 ± 32.1 N/mm^2^) ligaments. The iliofemoral ligament (53.5 ± 15.1 mm^2^) yielded a significantly higher cross section compared to the ischiofemoral (19.2 ± 13.2 mm^2^) and pubofemoral (15.2 ± 7.2 mm^2^) ligament. No significant gender- or side-specific differences were determined. A comparison to the published data on the iliotibial tract revealed lower elasticity and less variation in the ligaments of the hip joint.

**Conclusion:**

Comparison of the mechanical data of the hip joint ligaments indicates that their role may likely exceed a function as a mechanical stabilizer. Uniaxial testing of interwoven collagen fibers might lead to a misinterpretation of the mechanical properties of the hip capsule ligaments in the given setup, concealing its uniaxial properties. This underlines the need for a polyaxial test setup using fresh and non-embalmed tissues.

## Introduction

Beside the stability-maintaining configured bony shape, both the passive stabilizers (hip capsule and ligaments) and active stabilizers (external rotators, the gluteal muscles and the rectus femoris) preserve hip stability [1]. The hip capsule ligaments strengthen the hip capsule and are merged with the fibrous part of the hip capsule. Thus, only the synovial part of the hip capsule can be removed without destroying the hip capsule ligaments [2]. For the presented study we therefore used the term “hip capsule ligaments” intending to also investigate the merged fibrous part of the hip capsule. The hip capsule ligaments, namely the iliofemoral ligament (IF), ischiofemoral ligament (IS) and pubofemoral ligament (PF), are known to guide and restrict the maximum possible range of motion and translation increasing hip joint stability [3,4]. Transecting or venting these ligaments, as done in arthroplasty or arthroscopy, enhances complications such as dislocations or iatrogenic instability, further illustrating the pivotal role of the hip joint ligaments [5–11]. Hence, more detailed knowledge on the mechanical properties of the hip joint ligaments are of legitimate interest.

Recent reports on the mechanical properties of the hip capsule ligaments yielded varying results, especially lacking in data pertaining to the PF ligament [12–14]. Trials on gender-specific differences have to date not been performed, though clinically higher capsular laxity was described in female patients possibly affecting the mechanical properties [9,15–17]. Previously, data of the mechanical properties were compared to the shoulder capsule possibly leading to a misinterpretation of their impact in joint stability [12,13,18,19]. Actually, the hip capsule ligaments have to date not yet been compared to other passive hip stabilizers in the pelvic ring region like the iliotibial tract. In the given study, our group obtained stress-strain values of human IF, IS and PF ligaments using a similar setup as used in previous studies [20,21]. Addressing these issues, we investigated the following hypotheses:

1a) The IF, IS and PF ligaments differ in their cross-sectional areas, strain, and elastic modulus.
1b) IF, IS and PF ligaments have side- and gender-dependent mechanical properties.
2) Stress-strain data of the hip capsule ligaments are comparable to the iliotibial tract because both passively contribute to hip joint stability.

## Materials and Methods

### Ethical statement

All tissue samples were obtained from human body donors who gave their signed consent before passing away for the use of their cadavers for research and educational purposes. Institutional approval was obtained and tissues were used in accordance to the Saxonian Death and Funeral Act of 1994 (S1 Table). Signed body donor consents are available on reasonable request from the senior author (H.S.).

### Tissue preparation

Ethanol-glycerin embalmed hip capsules with adjacent ligaments were obtained from 17 cadavers (9 females [F], 7 males [M], 13 from the left [L] and 8 from the right [R] side, mean age 83.65 ± 10.54 years; Table 1) [20,22,23]. The hip joint was dissected by removing the surrounding soft tissue, presenting the complete hip capsule. Medical students completed this part of the dissections during their anatomical dissection course. The authors completely removed the hip capsule ligaments from the acetabular rim and the femoral attachments, namely the intertrochanteric line and the lesser trochanter, after placing orientation markings. The synovial part of the hip capsule was removed. The ligaments of the hip capsule including the fibrous part of the synovial membrane were tested in this setup [2]. Then the directed ligaments of the hip capsule were identified and divided, based on their superficial fiber orientation. Using this approach, we obtained the inferior portion of the IF ligament, which was subsequently named as IF ligament. We cut smaller probes of obviously superficial fibers from the parts of the IF, IS and PF ligaments to assure the test of the whole probe and reducing non-orientated parts. After dissection, 18 IF, 9 IS and 17 PF ligaments were used for stress-strain data generation. After dissection the ligaments of the hip capsule were stored in the conservation fluid consisting of water-diluted ethanol and glycerin and after hydration frozen by -80°C [23]. To minimize the dehydrating effects of the ethanol fixation with alterations of the elastic modulus as a consequence, the tissues were washed in isotonic sodium chloride solution for 24 hours before further procedures [20,23].

**Table 1:** Baseline data of the hip joint capsule specimens. Mean values ± standard deviation are given in the captions for all tested tissue samples (∑).

Tissue number	Age [years]	Gender	Side	Cause of death
Σ = 17	83.65 ± 10.54	F:M (9:7)	L:R (13:8)	
1	95	F	L	Chronic cardiac failure
2	78	M	L	Acute myocardial infarction
3	97	F	L	
4	85	F	R	Pneumonia
5	79	F	L	Pulmonary embolism
			R	
6	89	F	L	Pneumonia
7	68	M	L	Pulmonary embolism
			R	
8	104	F	L	Chronic cardiac failure
9	67	F	L	Acute pancreatitis
10	74	M	L	Heart-lung failure
			R	
11	87	M	R	Chronic cardiac failure
12	79	F	L	Cardiac failure
			R	
13	89	M	R	Cardiac failure
14	84	M	L	Pneumonia
			R	
16	71	M	L	Unclear
17	81	F	L	Cardiac failure

F = female, M = male, L = left and R = right.

### Partial plastination of tissue endings

**Fig 1:**
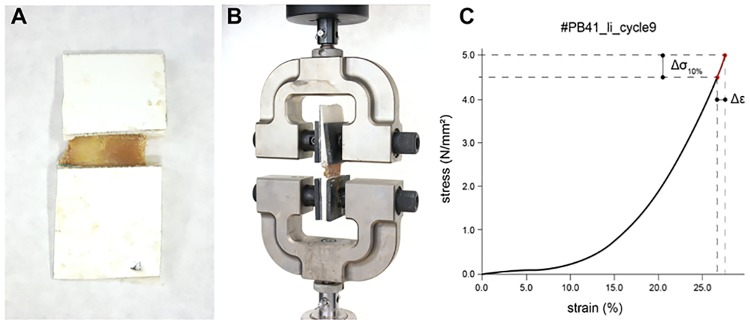
Preparation and material testing setup for the investigation of the stress-strain properties of the ilio-, ischio- and pubofemoral ligaments. (A) Partially plastinated ligaments of hip capsule. (B) Attached partially-plastinated tissue sample to the testing machine (C) Example of a stress-strain curve. The elastic modulus was represented by means of a secant modulus. It was calculated as ratio of Δσ10% (last 10% of stress values) and according strain values Δε: elastic modulus = Δσ10% / Δε.

The ends of the respective hip capsule ligaments were partially plastinated according to the published protocol (Fig 1A) [20,21]. In brief, the ligaments’ ends were substituted with acetone under freezing conditions and treated with a resin. Covering the ligaments’ ends with Pertinax plates (PF CP 201, Dr. Müller GmbH, Ahlhorn, Germany) and aluminum blocks reinforced the stability of plastination, facilitated resin penetration and created a plane area without plastination [20,21]. The aluminum blocks were covered with silicon oil to release the tissue after polymerization. The ends of the tissue were immersed in resin, a vacuum was applied to extrude the acetone and the resin entered the tissue. By subjecting the tissue to 40°C-warm water the gelatin melted and the aluminum blocks were released from the unplastinated area.

### Mechanical testing

The tissue was stored in isotonic sodium chloride solution until and sprinkled during the uniaxial tensile testing. The mechanical tests were performed using an Instron testing machine (5566A Dual Column Table-Top Frame Testing Machine, Norwood, USA) under room temperature conditions (Fig 1B).

The tests were performed with a crosshead displacement velocity of 5 mm/min. The 1 kN load cell had a measurement accuracy of ± 0.4% between 1 and 100% of the load capacity and an accuracy of ± 0.5% between 0.25 and 1% of the load capacity. In an initial speed-controlled trial, the minimum force of plastic deformation was determined from representative samples (seen in the load-deformation graphs or macroscopically visible signs of failure; own unpublished results). In the consecutive testing cycles, the force maximum was set at 90% of the minimum force causing plastic deformation from the previous trial. Furthermore, to avoid plastic deformation of the ligament samples, a displacement rate of 12 mm/min was chosen in a static range. Hence, the hip capsule ligaments were tested in a pre-determined range of elastic deformation and failure was not investigated to reduce failure related decreases in the subsequent determined cross section. The load-deformation curves were recorded displaying a linear gradient in the investigated range. Based on these curves, the elastic modulus was computed as a secant modulus of the most linear region. The secant modulus of each cycle of each tissue sample was then calculated from the data in the final region of the stress-strain curves as presented in Fig 1C. After the biomechanical tests, the ligaments were marked externally by placing metal clamps at the respective levels of the least cross section, thereby indicating the level where the cross sections was measured. The samples, including the metal clamps, were then fully plastinated and cut perpendicularly to the ligament fiber direction, guided by the metal clamps. The cross-sectional areas were scanned at 1200 dpi (HP Scanjet, Palo Alto, USA) and the cross sections were measured at least two times by one investigator (Datinf GmbH, Tübingen, Germany; S2 Table).

### Data evaluation

The recorded data of each test cycle of each sample were used for evaluation. The strain and the elastic modulus were calculated as the mean value of the ten consecutive test cycles. Pooling these individual mean values, the general mean values ± standard deviations (SD) were generated for the hip capsule ligaments (IF, IS, PF). The mean values ± SD of the cross sections were derived from pooling the mean values of the two measurements (P.P, T.W.). Graphs were plotted and analyzed using Graph Pad Prism software 6 (La Jolla, USA). Testing the values regarding Gaussian distribution was performed using Shapiro-Wilk test yielding a non-Gaussian distribution for strain, cross section and elastic modulus. For investigations of side- and gender-specific differences a non-Gaussian distribution was presumed related to the small sample size. Hence, for statistical analyses the Kruskal- Wallis with a Dunn´s multiple comparison test were performed. For comparison of the iliotibial tract to the hip capsule ligaments the Mann-Whitney U test was chosen. The same statistical tests were used investigating gender- or side-specific differences. Significance level was set to p values of 0.05 or less.

For the comparison to the iliotibial tract, elastic modulus data were used as a secant modulus in a similar setup as used here for the hip capsule ligaments on basis of the values published previously by our group [21]. Here, the data in the range from 4 to 11 N/mm^2^ of the old donor group were used related to the reported linear course of the stress-strain data indicating an elastic behavior. The setup including partial plastination and the same testing environment was used to assure comparable results.

## Results

During the dissection, we recognized an incongruent fiber orientation between the superficial and profound layers of the hip capsule ligaments. This observation was proven in hip joint plastinates cut in different orientations obtained from medical courses (S1 Fig). The non-orientated parts of the hip capsule could not be assigned to the ligaments and were thus not tested. During testing, one IF ligament failed without any signs of non-linear deformation within the stress-strain data. This sample was excluded from further investigations. The data were generated from 17 IF, 9 IS and 17 PF ligaments obtained from the linear part of the curves.

### The IF ligament showed a significantly larger cross section compared to the IS and PF ligament

**Fig 2:**
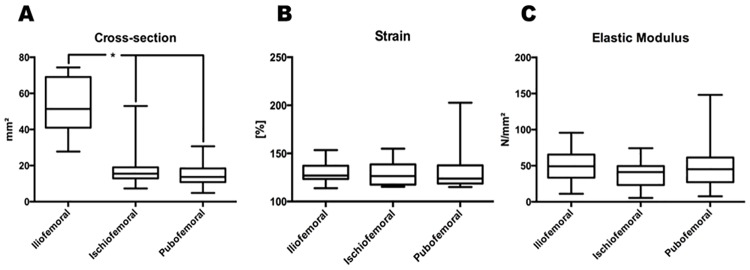
The ligaments of the hip capsule revealed similar mechanical properties except of significant differences in the cross-sectional area. Hip capsules of 17 cadavers were used and dissected related to their anatomical origin. From these tissue samples 17 ilio-, 9 ischio- and 17 pubofemoral were investigated regarding their elastic modulus, strain and cross-sectional area. (A) The cross-section of the iliofemoral ligament (IL) is significant higher compared to ischio- (IS) or pubofemoral (PF) ligament (* indicates p<0.05 adjusted p values: IF vs. IS 0.0016, IF vs. PB <0.0001, IS vs. PB >0.999). (B) Analysis of strain values revealed non-significant differences between the hip capsule ligaments. (C) Elastic Modulus was generated related to the strain and stress values measured over ten cycles and present no significant differences between iliofemoral, ischiofemoral and pubofemoral ligament.

We determined a cross-sectional area of 53.5 ± 15.1 mm^2^ for the IF ligament, 19.2 ± 13.2 mm^2^ for the IS- and 15.2 ± 7.2 mm^2^ for the PF ligament (Fig 2A, S2 Table). Comparing the cross-sectional areas yielded a significantly larger cross-sectional area of the IF ligament compared to IS- and PF ligament (p<0.05; adjusted p values: IF vs. IS: p = 0.0016, IF vs. PB: p<0.0001). However, the cross-sectional area between the IS- and PF ligament did not presented significant differences (IS vs. PB: p>0.99).

### Strain of the hip capsule ligaments were similar and non-significantly different

In accordance to the above-mentioned procedure we determined the following strain data:

Mean Strain: 129.8 ± 11.1% for the IF ligament, 128.7 ± 13.7% for the IS ligament and 133.2 ± 23.7% for the PF ligament (Fig 2B, S3 Table). No significant differences between the hip capsule ligaments regarding strain were determined (p>0.99 for each comparison).

### The elastic moduli of the hip capsule ligaments were similar without significant differences

Elastic moduli were computed as follows: 48.8 ± 21.4 N/mm^2^ for the IF ligament, 37.5 ± 20.4 N/mm^2^ for the IS ligament, and 49.0 ± 32.1 N/mm^2^ for the PF ligament (Fig 2C, S2 Table). Statistical analyses yielded no significant differences between the hip capsule ligaments (p>0.05, adjusted p values: IF vs. IS: p = 0.64, IF vs. PB: p>0.99, IS vs. PB: p = 0.98).

### Strain, elastic modulus and cross section of the hip capsule ligaments displayed no gender- or side-dependent differences

Comparing the strain, elastic modulus and cross section the obtained values regarding gender-dependent differences displayed no significant differences (Table 2; p>0.99 for cross section, strain and elastic modulus). The following values were obtained:

Strain:
  - 131.5 ± 14.96% male iliofemoral (M-IF)- vs. 128.3 ± 6.64% female iliofemoral (F-IF) ligament,
  - 126.3 ± 12.21% male ischiofemoral (M-IS)- vs. 131.6 ± 16.71% female ischiofemoral (F-IS) ligament and
  - 135.1 ± 20.02% male pubofemoral (M-PF)- vs. 131.6 ± 27.75% female pubofemoral (F-PF) ligament.
Elastic modulus:
  - 53.43 ± 27.49 N/mm^2^ M-IF- vs. 44.69 ± 14.72 N/mm^2^ F-IF ligament,
  - 42.38 ± 20.43 N/mm^2^ M-IS- vs. 31.33 ± 21.56 N/mm^2^ F-IS ligament and
  - 48.925 ± 23.66 N/mm^2^ M-PF- and 49.14 ± 39.59 N/mm^2^ F-PF ligament.
Cross section:
  - 55.07 ± 17 mm^2^ M-IF- vs. 52.07 ± 14.13 mm^2^ F-IF ligament,
  - 20.8 ± 18.29 mm^2^ M-IS- vs. 17.27 ± 3.34 mm^2^ F-IS ligament and
  - 14.06 ± 5.67 mm^2^ M-PF- vs. 16.21 ± 8.523 mm^2^ F-PF ligament.

Side-specific examinations revealed the following values:

Strain:
  - 129 ± 9.03% right iliofemoral (R-IF)- vs. 130.2 ± 12.45% left iliofemoral (L-IF) ligament,
  - 126.3 ± 8.03% right ischiofemoral (R-IS)- vs. 129.8 ± 16.39% left ischiofemoral (L-IS) ligament and
  - 145.5 ± 36.79% right pubofemoral (R-PF)- vs. 126.6 ± 9.31% left pubofemoral (L-PF) ligament.
Elastic modulus:
  - 64.63 ± 18.87 N/mm^2^ R-IF- vs. 40.16 ± 18.01 N/mm^2^ L-IF ligament,
  - 18.21 ± 11.13 N/mm^2^ R-IS- vs. 47.09 ± 16.85 N/mm^2^ L-IS ligament and
  - 39.89 ± 19.82 N/mm^2^ R-PF- and 54.02 ± 37.04N/mm^2^ L-PF ligament.
Cross section:
  - 43.29 ± 11.93 mm^2^ R-IF- vs. 59.03 ± 14.09 mm^2^ L-IF ligament,
  - 26.53 ± 23.7 mm^2^ R-IS- vs. 15.58 ± 2.71 mm^2^ L-IS ligament and
  - 16.2 ± 10.89 mm^2^ R-PF- vs. 14.66 ± 4.73 mm^2^ L-PF ligament.

**Table 2:** Investigation of gender-specific differences of the hip capsule ligaments regarding strain, cross section and elastic modulus. No statistical differences were determined (p>0.05).

	Iliofemoral (n = 17)		Ischiofemoral (n = 9)		Pubofemoral (n = 17)	
	*Female (n = 9)*	*Male (n = 8)*	*Female (n = 4)*	*Male (n = 5)*	*Female (n = 9)*	*Male (n = 8)*
Strain [%]	128.3 ± 6.64	131.5 ± 14.96	131.6 ± 16.71	126.3 ± 12.21	131.6 ± 27.75	135.1 ± 20.02
Cross section [mm^2^]	52.07 ± 14.13	55.07 ± 17	17.27 ± 3.34	20.8 ± 18.29	16.21 ± 8.52	14.06 ± 5.67
Elastic modulus [N/mm^2^]	44.69 ± 14.72	53.43 ± 27.49	31.33 ± 21.56	42.38 ± 20.43	49.14 ± 39.59	48.92 ± 23.66

Side-specific investigations revealed no significant differences (Table 3, p>0.99 for cross section and strain, elastic modulus R-IF vs. L-IF: p = 0.55, R-IS vs. L-IS p = 0.61, R-PF vs. L-PF: p>0.99).

**Table 3:** Investigation of side-specific differences of the hip capsule ligaments regarding strain, cross section and elastic modulus. No Statistical differences were determined (p>0.05).

	Iliofemoral (n = 17)		Ischiofemoral (n = 9)		Pubofemoral (n = 17)	
	*Left (n = 11)*	*Right (n = 6)*	*Left (n = 6)*	*Right (n = 3)*	*Left (n = 11)*	*Right (n = 6)*
Strain [%]	130.2 ± 12.45	129 ± 9.03	129.8 ±16.39	126.3 ± 8.03	126.6 ± 9.31	145.5 ± 36.79
Cross section [mm^2^]	59.03 ± 14.09	43.29 ± 11.93	15.58 ± 2.71	26.53 ± 23.7	14.66 ± 4.73	16.2 ± 10.89
Elastic modulus [N/mm^2^]	40.16± 18.01	64.63 ±18.87	47.09 ± 16.85	18.21 ± 11.13	54.02 ± 37.04	39.89 ± 19.82

### The ligaments of the human hip capsule were more elastic and showed less variation than the human iliotibial tract

**Fig 3:**
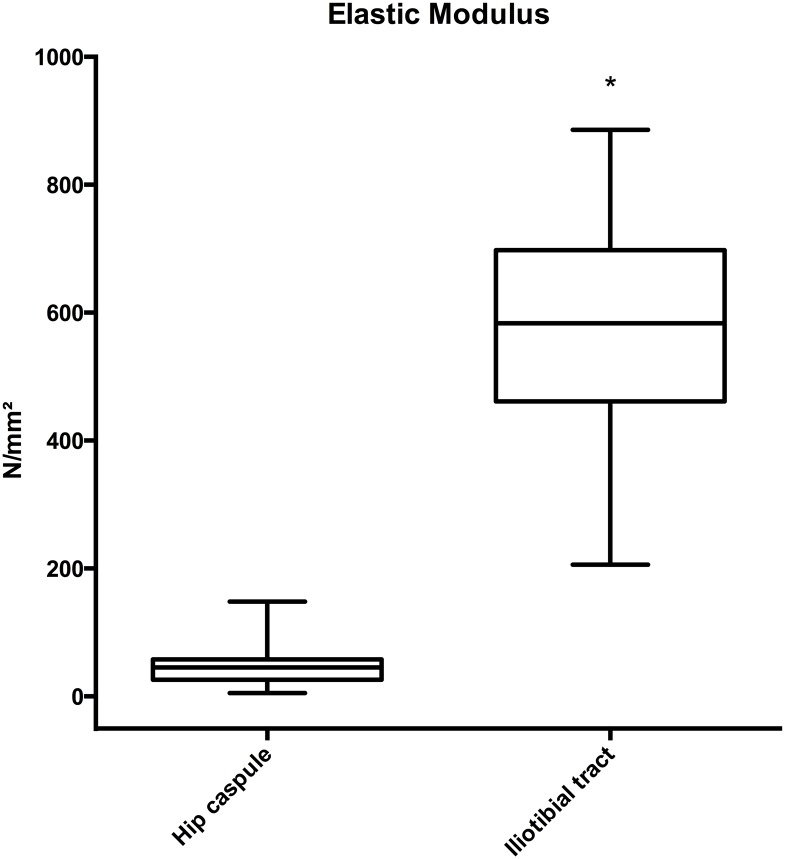
The ligaments of the hip joint capsule have a lower elasticity and less variation compared to the iliotibial tract (* indicates p<0.0001).

Based on the reported elastic behavior of the iliotibial tract, indicated by the linear course of stress-strain data by Hammer et al. 2012 [21], the values within the range from 4–11 N/mm^2^ (old donor group) were compared to the given data of the hip capsule ligaments. The mean elastic modulus of the hip capsule ligaments (46.5 ± 26.0 N/mm^2^) was significantly lower than for the iliotibial tract (564.7 ± 193.8 N/mm^2^), respectively (Fig 3; p<0.0001).

## Discussion

### Comparison with previous biomechanical tests of the hip capsule ligaments revealed varying results with implications for the test setup

In recent studies the stress-strain data, cross section and elastic modulus of the hip capsule and their ligaments were investigated yielding varying results [12–14]. Hewitt et al. investigated the hip capsule ligaments (n = 10 IF and n = 10 IS ligaments) in a fresh-frozen bone-ligament-bone interface [12,13]. They found a significantly higher elastic modulus and cross section for the IF compared to the IS ligament. We found this only for the cross section. Comparing the here-obtained data with the data of Hewitt et al. [12,13], our data revealed lower stress-strain values. Furthermore, data of the PF ligament were not determined. Yet, not only the IF and IS ligament contribute to hip stability. Therefore Stewart et al. investigated the material properties of the intact capsule (n = 10) and subsequently of eight parts cut parallel to the superficial fiber orientation [14]. Here, the authors did not state that the ligaments were separately removed, suggesting that they used the terminus hip capsule for the hip capsule and its surrounding ligaments. Hence, the hip capsule ligaments, the fibrous and the synovial part of the hip capsule were examined. The authors did not find any significant differences between the investigated tissue samples. Obtained data of Stewart et al. [14] revealed values that are smaller than Hewitt et al.'s values [12,13]. Indeed, we did not used fresh-frozen tissue samples but the ethanol-glycerin fixation method is known to only marginally affect the elastic modulus [20,22,23]. After watering the samples with isotonic sodium chloride solution, the alterations of the fixation method are clearly reduced [20]. In addition, the small sample size of all studies, including the present study, might be a limitation leading to such varying results. A further explanation for the differing results might also be present in the different test setup. Where Hewitt et al. used a bone-ligament-bone interface, measuring not only the hip capsule ligaments but also the adjacent bone, and Stewart et al. measured the eight samples of the hip capsule possibly destroying the fiber structure. As known from histological investigations, the IF fibers are mainly orientated parallel to one another, whereas the IS displayed a big amount of fibers perpendicular to the superficial orientation [24]. The varying fiber orientations within the hip capsule ligaments might reveal an attempt to explain the differing results of Hewitt et al., Stewart et al. and the presented data here. Additionally, in these studies, specimen dimensions might have differed with effect onto the stress-strain values. Thus a difference of damaged deeper collagen layers, possibly not parallel to the superficial orientation, of the hip capsule ligaments might be expected. Hence, these layers and fibers cannot be measured resulting in an underestimation of the biomechanical properties of examined sampled independent of length and cross section, e.g. for the IS. We conclude from our data that the hip capsule ligaments in total are more elastic than suggested by Hewitt et al. [12,13]. The difference between our data and that of Hewitt et al. might be explained by the examination of larger tissue samples by Hewitt´s group. This might be related to testing deeper collagen layers and fibers which are not in the supposed testing direction. Considering the bias, a polyaxial test setup is needed to get realistic insights into the mechanical behavior of the hip capsule ligaments related to the layers formed like a wire mesh (S1 Fig). Nevertheless, we assume our data to be reliable to generate baseline data due to the decreased material slippage [12–14,25]. It needs to be taken into account that ethanol-fixed tissue samples were used and that time has elapsed until the cadavers were cooled and embalmed. Although tendons, aortic, muscle and spine samples are different from ligaments, for these tissues no significant alterations were reported, especially in the first three days [26–29]. Furthermore, it could be shown by others that the stress-strain and failure-load data were minutely affected by post mortem delay up to 96 hours in a rabbit model [30]. Nevertheless, the effects of water content, drying and fixation, as known from Thiel fixation, cannot completely excluded and thus limit the estimation of a realistic behavior [31–33].

Beside this issue, with respect to the histological architecture of the hip joint ligaments, also the differences in data of the anatomical course and related preparation are challenging determining realistic material properties of the hip capsule ligaments [34]. Although the anatomical course of the hip capsule ligaments seems to be completely elucidated, reports differ. This could be related to the interwoven structures of the ligaments or to individual findings in anatomical specimens and patients [4,34–36]. In addition, the thickness of the hip capsule and their ligaments might vary as a result of pathological alterations like femoroacetabular impingement (FAI) also affecting the data [37,38]. Apart from these limitations, we have to discard our hypothesis that the IF-, IS- and PF ligament differ in strain and elastic modulus.

### The clinical importance of the hip capsule and its ligaments

Following hip joint surgery, such as arthroplasty (THA) or arthroscopy, the detrimental effects of transecting the hip capsule ligaments are revealed by decreasing stability and increased dislocation events [3,5–8,39,40]. Studies were performed investigating the effect of the surgical approach on hip stability, yielding it as an important factor influencing hip joint stability [5,7]. Related to the approach, different parts of the hip capsule and capsule ligaments were dissected and either left open or reconstructed, initiating an extensive discussion of the role of capsular reconstruction, where capsular reconstruction seems to be beneficial [7]. In addition, it was shown that the surgical approach predicts the dislocation direction of the hip prosthesis [41]. Thus, the whole hip capsule ligaments might be involved in reducing dislocation events. Hence, the ability of hip capsule ligaments do not differ, as supported by our data and contradicting previously obtained data [12–14]. The suggestion of the beneficial effect of capsular reconstruction is supported by some finite element studies, based on the data of Hewitt et al., confirming the importance of the hip capsule and its ligaments [5–8,12,13]. Also, in hip arthroscopy, the hip capsule and its ligaments are of pivotal interest [9–11]. Capsular laxity was associated to labral tears and injuries leading to an elongation of the IF ligament [11]. Due to increasing hip arthroscopy procedures, the recognized iatrogenic hip instabilities increased [9,42]. Thus, McCormick et al. reported about 1.23% revisions within one year due to labral injuries and capsular insufficiency. In these cases magnetic resonance imaging (MRI) revealed an abnormal capsular structure without indications of FAI. All these patients did not have a capsular repair in their first arthroscopy. These studies underline the importance of the hip capsule ligaments and lead to the question how much the mechanical properties contribute to their stabilizing effects.

### Gender- and side-dependent differences of the hip capsule ligaments

Apart from the question, how much impact the mechanical properties have in hip stability, previous studies offered evidence for gender-specific differences in the capsular behavior by the observation of longer hip capsules in women compared to hip capsules of men [37]. In contrast to the length, men displayed a bigger capsular volume in MRI studies [43]. Further evidence for gender-specific alterations of the stress-strain properties are given by the observations on capsular insufficiency predominantly in female patients and hip laxity found especially in female cadavers [3,9]. An explanation for these observations is currently not available. Data from investigations of the collagen content and effects on ligament healing in relation to sexual hormone receptors offered evidence for gender-specific mechanical properties and might reveal an attempt at explanation for the clinical observations [15–17]. In our test setup we were not able to determine gender-specific differences. These missing differences might be due to the attrition of ligaments, to the postmenopausal state of the female cadavers and related decreases in sexual hormones such as estrogen and to missing data to the weight bearing of the cadavers. Based on the predominant unilateral occurrence of pathologies like hip laxity side-specific differences are possible and suggested to better describe the hip joint behavior [12]. Nevertheless, in our study we were not able to determine such differences suggesting that side-specific differences of the hip capsule ligaments do not contribute to the occurrence of such pathologies. But so far, the affection of these properties might be changed as consecutive event to these pathologies as seen in variations of hip capsule size and thickness [37,38].

### Comparison of the mechanical properties of the hip capsule ligaments with other ligamentous tissue

In recent studies the mechanical properties of the hip capsule were compared to the shoulder capsule and on this basis graded as pivotal stabilizer [13]. A comparison to an adjacent passive hip stabilizer, namely the iliotibial tract, is in our view preferable, because the iliotibial tract is known to transfer high loads [44]. Thus, we compared the elastic modulus of the hip capsule ligaments with previously- obtained data of the iliotibial tract [21]. The hip capsule ligaments were less stiff and their stiffness showed less variation when compared to the iliotibial tract. In contrast to the hip capsule ligaments, the iliotibial tract consists of mainly parallel-aligned collagen fiber bundles [44,21]. In our view, the interwoven structures of the hip capsule ligaments might be necessary to preserve an elastic hip stability. However, if the system is in pre-load, it could be rigid, like a tensioned wire mesh. This leads to the assumption that not only the mechanical force transduction of the hip capsule contributes to hip stability, especially regarding hip arthroscopy and the occasional small defects. The effect of capsular release might be due to a loss of the synergistic effects of passive and active stabilizers (external rotators, the gluteal and rectus femoris muscles), as known from the concept of form and force closure of the sacroiliac joint [1,45–49]. Although the majority of patients displayed at least proximal or distal contact after capsule closure in hip arthroplasty, the mechanical behavior of the neo-capsule might not be comparable to the preoperative capsule as seen in their re-organized structure [7,50,51]. Hence, the natural repair of the capsule might also have other implications. Other theories were established such as the reduction of cavities surrounding the implant neck avoiding fluid-filled rooms as an elastic mechanical block. In the hip capsule a sealing was seen to obtain a pressure dependent stability [6,52–57]. Our values underline such theories. The lower elastic moduli calculated from our data support the hip capsule ligaments to fulfill such functions. This may also explain the merging and strengthening of the fibrous part by the IF-, IS- and PF ligament. However, it needs to be mentioned that our study has a number of limitations. By investigating exclusively elderly cadavers originating and in a very limited sample size [33] we are unable to determine the potential effects of aging, which are well known to alter the mechanical properties of the hip capsule [12–15,17,18,24,34,46]. We also did not use a polyaxial test setup, which would have been necessary to measure a wire-mesh-like collagen arrangement as presumably existent in the capsular ligaments of the hip joint [2]. Another potential bias of the data was introduced measuring the middle part of the ligament only, which is known to be weaker than its attachments [31]. Another drawback of the present study is the missing separation of the hip capsule ligaments and the fibrous part of the hip capsule [2]. Thus, the damage resulting from different preparation techniques in relation to the subsequently used test setup might result in the differing data presented by Hewitt et al., Stewart et al. and the present study [12–14]. In addition, the fixation by dehydration with ethanol and acetone and the post mortem delay have affected the data [20,22,23,31]. Furthermore, our mechanical data are limited by the missing data of ultimate stress. Thus, the elastic properties of the hip capsule ligaments may be overestimated. Although the displacement rates were chosen in a static range in the given study to allow for comparability to previous studies and to examine the samples externally during the mechanical testing, micro-damage of the samples cannot be excluded. These limitations might be an attempt at explanation of the differences to the previously-obtained data of the hip capsule ligaments [12–14] In future, data obtained in a fresh and non-embalmed condition, also from younger and in a larger sample size may be of interest so substantiate our findings.

## Summary

The obtained elastic modulus of the hip capsule ligaments was lower compared to the previously- obtained elastic modulus of the iliotibial tract and presented data lie in between previously-performed studies [12–14]. We found no gender- and side-specific differences in the hip capsule ligaments. Discussing our results with previous studies, polyaxial testing of the ligaments of the human hip joint are necessary to address the different fiber orientations. Our values indicate that the hip-stabilizing effect of the ligaments might not only be due to their involvement in load transfer. There seems to be an interaction between the passive and active hip stabilizers. If the capsule is as elastic as our data suggest, the capsule may be adhere to the joint without high tension during hip movement. Thus, we conclude that the hip capsule together with its (elastic) ligaments functions like a cuff that is tautened by the active stabilizers of the hip. Therefore, we reject the hypothesis that the mechanical properties of the hip capsule are solely responsible for the stabilizing effect of the hip capsule to hip stability.

## Supporting Information

S1 Fig Plastinated axially orientated hip joint cut along the femoral neck obtained from medical student course yielding the interwoven fibers of the hip capsule ligaments, indicated by *. (TIF)

S1 Table Saxonian Death and Funeral Act. (PDF)

S2 Table Strain [%] and stress [N/mm^2^] data of hip capsule parts of the anatomical sites: iliofemoral, ischiofemoral and pubofemoral. Data display the mean, minimum and maximum values measured over all ten test cycles. F = female, M = male, L = left and R = right.
(PDF)

S3 Table Measured cross section [mm^2^] and derived elastic modulus [N/mm^2^] of the hip capsule ligaments separated related to their anatomical origin. Mean values ± standard deviations for every ligament are presented in the caption. F = female, M = male, L = left and R = right.
(PDF)
