# Supplementary figures and images for: The Stress-Strain Data of the Hip Capsule Ligaments Are Gender and Side Independent Suggesting a Smaller Contribution to Passive Stiffness
Source: PLoS One. 2016 Sep 29;11(9):e0163306. doi: 10.1371/journal.pone.0163306 (PMC5042535; doi:10.1371/journal.pone.0163306)

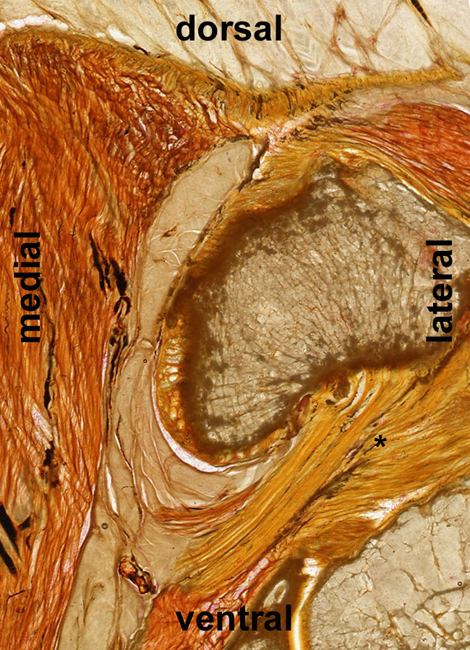

Supplement: S1 Fig — (TIF) [file pone.0163306.s001.tif]
