# Supplementary material for: The Stress-Strain Data of the Hip Capsule Ligaments Are Gender and Side Independent Suggesting a Smaller Contribution to Passive Stiffness
Source: PLoS One. 2016 Sep 29;11(9):e0163306. doi: 10.1371/journal.pone.0163306 (PMC5042535; doi:10.1371/journal.pone.0163306)
Supplement: S1 Table — (PDF) [file pone.0163306.s002.pdf]

Sächsisches Gesetz über das Friedhofs-, Leichen- und Bestattungswesen vom 8. Juli 1994

Stand: 01.03.2014
letzte berücksichtigte Änderung: geändert durch Artikel 8 des Gesetzes vom 13. Dezember 2012 (SächsGVBl. S. 725, 731).

Der Sächsische Landtag hat am 23. Juni 1994 das folgende Gesetz beschlossen:

Erster Abschnitt

Friedhofswesen

§ 1
Bestattungsplätze

(1) Bestattungsplätze sind

1. Gemeindefriedhöfe,
2. Friedhöfe der Kirchen und Religionsgemeinschaften sowie Grabstätten in Kirchen,
3. Anstaltsfriedhöfe und sonstige private Bestattungsplätze.

(2) [1]Bestattungsplätze müssen der Würde des Menschen, den allgemeinen sittlichen Vorstellungen und den anerkannten gesellschaftlichen Ordnungen entsprechen. [2]Sie müssen so beschaffen sein, dass die Totenruhe gewährleistet und das Grundwasser sowie die Oberflächengewässer, die öffentliche Sicherheit sowie die Gesundheit und das Wohl der Allgemeinheit nicht beeinträchtigt werden. [3]Die Anforderungen einer geordneten städtebaulichen Entwicklung sowie die Belange der Landschafts- und Denkmalpflege sind zu berücksichtigen.

(3) [1]Die Neuanlage und die Erweiterung eines Bestattungsplatzes sowie die Wiederbelegung eines vorher geschlossenen Bestattungsplatzes bedürfen einer schriftlichen Genehmigung. [2]Sie ist zu erteilen, wenn die Voraussetzungen dieses Gesetzes erfüllt sind und sonstige Vorschriften des öffentlichen Rechts nicht entgegenstehen. [3]Zuständige Genehmigungsbehörde ist

1. in kreisangehörigen Gemeinden der Landkreis,
2. in Kreisfreien Städten die Kreisfreie Stadt.

(4) Vor der Erteilung der Genehmigung hat die Genehmigungsbehörde eine gutachtliche Stellungnahme des Sächsischen Landesamtes für Umwelt, Landwirtschaft und Geologie zu den geologischen und hydrogeologischen Gegebenheiten einzuholen und sich mit dem zuständigen Gesundheitsamt ins Benehmen zu setzen.

§ 2
Gemeindefriedhöfe

(1) Den Gemeinden obliegt es als Pflichtaufgabe, Friedhöfe anzulegen und zu erweitern sowie Leichenhallen zu errichten, soweit hierfür ein öffentliches Bedürfnis besteht, und diese Einrichtungen zu unterhalten. Diese Pflicht umfasst auch die Sorge dafür, dass die notwendigen Bestattungseinrichtungen zur Verfügung stehen.

(2) Auf Gemeindefriedhöfen ist die Bestattung verstorbener Gemeindeeinwohner zuzulassen. Die Bestattung anderer Verstorbener kann durch Satzung der Gemeinde ermöglicht werden. Die Bestattung einer anderen in der Gemeinde verstorbenen oder tot aufgefundenen Person ist außerdem zuzulassen, wenn diese keinen festen Wohnsitz hatte, ihr letzter Wohnsitz unbekannt ist, ihre Überführung an den früheren Wohnsitz unverhältnismäßig hohe Kosten verursachen würde oder wenn Gründe der öffentlichen Sicherheit oder Ordnung eine Bestattung in der Gemeinde erfordern.

(3) Auf Gemeindefriedhöfen sind in ausreichendem Umfang Reihengräber als Einzelgräber bereitzustellen. In der Benutzungsordnung der Friedhofsträger (§ 7 Abs. 1) ist zu regeln, in welchem Umfang andere Arten von Grabstätten, insbesondere Wahlgräber und Gemeinschaftsgrabanlagen, bereitgestellt werden und welche anderen Begräbnisformen zugelassen sind. 3

§ 3
Andere Friedhöfe und Bestattungsplätze

(1) Kirchen, Kirchgemeinden und Kirchgemeindeverbände sowie andere Religions- oder Weltanschauungsgemeinschaften, die Körperschaften des öffentlichen Rechts sind, können eigene Friedhöfe nach Maßgabe der Gesetze anlegen, erweitern und wiederbelegen (kirchliche Friedhöfe) sowie Leichenhallen errichten.

(2) Grabstätten in Kirchen der in Absatz 1 genannten Körperschaften sind als Bestattungsplätze zu genehmigen, sofern nicht durch die Bestattung im Einzelfall gesundheitliche Gefahren zu befürchten sind.

(3) Anstaltsfriedhöfe und sonstige private Bestattungsplätze dürfen nur angelegt, erweitert oder wiederbelegt werden, wenn

1. ein besonderes Bedürfnis oder ein berechtigtes Interesse besteht,

2. eine würdige Gestaltung und Unterhaltung des Bestattungsplatzes während der Ruhezeit gesichert sind und

3. öffentliche Interessen oder schutzwürdige Belange Dritter nicht entgegenstehen.

(4) [1]Jede Bestattung auf sonstigen privaten Bestattungsplätzen, die nicht Anstaltsfriedhöfe sind, bedarf einer besonderen Genehmigung durch die nach § 1 Abs. 3 zuständige Behörde. [2]Die Genehmigung darf nur für die Beisetzung von Aschen Verstorbener erteilt werden. [3]Sie ist insbesondere zu versagen, wenn die Bestattung mit der jeweils geltenden Bebauungsplanung nicht vereinbar ist.

(5) Die Veräußerung von Grundstücken, auf denen sich Anstaltsfriedhöfe oder sonstige private Bestattungsplätze befinden, ist der Genehmigungsbehörde anzuzeigen.

§ 4
Friedhöfe nichtgemeindlicher Träger

(1) Auf nichtgemeindlichen Friedhöfen, außer jüdischen, sind die in § 2 Abs. 2 genannten Verstorbenen aufzunehmen, soweit in zumutbarer Entfernung keine gemeindlichen Friedhöfe bestehen. Dies gilt auch dann, wenn es sich um andersgläubige oder konfessionslose Verstorbene handelt. Diese sind nach Möglichkeit ohne räumliche Absonderung von anderen Grabstellen zu bestatten; die Nutzung der Leichenhalle ist für sie zuzulassen. In die Art und Weise der Bestattungs- und Totengedenkfeiern sowie in die Gestaltung der Grabstätten darf nur unter den Voraussetzungen des § 7 eingegriffen werden.

(2) Die Gemeinden haben sich an dem Kostenaufwand anderer Träger, die in ihrem Einzugsbereich einen Friedhof im Sinne des Absatzes 1 Satz 1 unterhalten, angemessen zu beteiligen, soweit die Kosten nicht durch Einnahmen aus den für die Nutzer zumutbaren Gebühren gedeckt werden können. Das gleiche gilt, wenn die gemeindlichen Bestattungsplätze nicht ausreichen und soweit der andere Friedhofsträger im Einverständnis mit der Gemeinde Bestattungsplätze für die Allgemeinheit zur Verfügung stellt. Das Nähere wird durch Vereinbarung zwischen der Gemeinde und dem anderen Friedhofsträger geregelt.

§ 5
Standort- und Abstandsregeln

(1) Friedhöfe sollen in ruhiger Lage, insbesondere nicht in unmittelbarer Nähe von verkehrsreichen Straßen, Eisenbahnen, Flug-, Sport- und Vergnü-

gungsstätten, Industrie- und Gewerbebetrieben sowie von Anlagen, die der militärischen Verteidigung dienen, angelegt werden.

(2) [1]Friedhöfe sollen verkehrsgünstig gelegen und mit öffentlichen Verkehrsmitteln erreichbar sein. [2]Für den ruhenden Verkehr sollen ausreichende und geeignete Parkflächen bereitgestellt werden.

(3) Friedhöfe können als Mittelpunktanlagen für mehrere Gemeinden oder Gemeindeteile angelegt werden.

(4) Friedhöfe sind nach außen durch Bäume, Sträucher, Zäune, Mauern, Erdwälle oder auf ähnliche Weise hinreichend abzuschirmen.

(5) [1]Der Grenzabstand zwischen Friedhöfen und Wohngebäuden einschließlich deren Nebenanlagen muss mindestens 35 m betragen. [2]Zu Gewerbe- und Industrieanlagen einschließlich deren Nebenanlagen ist ein Grenzabstand von mindestens 75 m einzuhalten. [3]Es können geringere Abstände zugelassen werden, wenn dies mit den nachbarlichen Belangen vereinbar ist und Ruhe und Würde des Friedhofs nicht wesentlich beeinträchtigt werden. [4]Die Entscheidung hierüber trifft in den Fällen des § 1 Abs. 3 die dort genannte Behörde. [5]Im Fall der Errichtung oder Änderung eines zu einem Friedhof benachbarten Bauvorhabens wird die Entscheidung nach Anhörung des Friedhofsträgers durch die zuständige Bauaufsichtsbehörde getroffen; bei genehmigungsfreien Vorhaben entscheidet die untere Bauaufsichtsbehörde.

(6) Die vorgeschriebenen Grenzabstände gelten nicht für die Abstände von bestehenden Friedhöfen zu Wohngebäuden oder gewerblichen Einrichtungen, die vor Inkrafttreten dieses Gesetzes errichtet worden sind.

§ 6
Ruhezeit

(1) Für einen Bestattungsplatz oder für Teile eines Bestattungsplatzes wird in der Genehmigung nach § 1 Abs. 3 im Benehmen mit dem Gesundheitsamt festgelegt, wie lange Grabstätten nicht erneut belegt werden dürfen (Mindestruhezeit).

(2) [1]Die Mindestruhezeit beträgt bei Fehlgeborenen und bei Leichen von Kindern, die tot geboren oder vor Vollendung des 2. Lebensjahres gestorben sind, 10 Jahre, im Übrigen 20 Jahre. [2]Für Aschen Verstorbener gelten die Ruhezeiten entsprechend.

(3) Der Träger des Bestattungsplatzes kann in der Benutzungsordnung (§ 7 Abs. 1) längere als die durch Gesetz oder Verordnung vorgeschriebenen Ruhezeiten vorsehen.

(4) Sofern die Benutzungsordnung (§ 7 Abs. 1) den Angehörigen des Verstorbenen ein Nutzungsrecht an der Grabstätte für die Dauer der Mindestruhezeit oder länger einräumt, handelt es sich um ein Nutzungsverhältnis öffentlich-rechtlicher Art.

(5) Während der Ruhezeit dürfen in einer Grabstätte weitere Leichen oder Aschen Verstorbener nur beigesetzt werden, wenn die Grabstätte dazu geeignet und bestimmt ist; das Nähere regelt die Benutzungsordnung (§ 7 Abs. 1).

(6) Die Ruhezeiten nach Absatz 2 gelten nicht für die bei Inkrafttreten dieses Gesetzes bereits belegten Grabstätten.

§ 6a
Ruherecht für Angehörige der Bundeswehr

(1) [1]Für Ehrengräber von Angehörigen der Bundeswehr, deren Tod bei oder infolge einer besonderen Auslandsverwendung im Sinne des § 63b des Gesetzes über die Versorgung für die ehemaligen Soldaten der Bundeswehr und ihre Hinterbliebenen (Soldatenversorgungsgesetz – SVG) in der Fassung der Bekanntmachung vom 16. September 2009 (BGBl. I S. 3054), das zuletzt durch Artikel 14 des Gesetzes vom 21. Juli 2012 (BGBl. I S. 1583, 1595) geändert worden ist, eingetreten ist, ist in der Benutzungsordnung (§ 7 Abs. 1) vorzusehen, dass das Grab auch nach Ablauf der Ruhezeit auf Dauer bestehen bleibt (dauerndes Ruherecht). [2]Das dauernde Ruherecht ruht als öffentliche Last auf dem Grundstück.

(2) [1]Die Der Friedhofsträger hat gegen den Freistaat Sachsen Anspruch auf Erstattung des mit dem dauernden Ruherecht entstehenden Vermögensnachteils. [2]Die Höhe bemisst sich nach der ortsüblichen Grabnutzungsgebühr und der Friedhofsunterhaltungsgebühr.

(3) Absatz 1 findet keine Anwendung, wenn der Tote in einer mehrstelligen Grabstätte (Wahl- oder Gemeinschaftsgrabanlage) bestattet ist, in der bereits ein Toter beigesetzt ist oder noch beigesetzt werden kann, dessen Grab nicht unter Absatz 1 fällt.

(4) [1]Die Gemeinde hat die auf ihrem Gebiet liegenden Gräber zu erhalten, es sei denn, es handelt sich um ein Grab, dessen Erhaltung Angehörige des Verstorbenen oder Dritte zeitweilig oder dauerhaft übernommen haben (privat gepflegtes Grab). [2]Maßnahmen der Erhaltung sind insbesondere die Instandsetzung und die Grabpflege. [3]Der Freistaat Sachsen erstattet der Gemeinde die notwendigen Aufwendungen für die Erhaltung der Gräber.

(5) Die Gemeinde hat auf Antrag der Angehörigen die Erhaltung eines privat gepflegten Grabes zu übernehmen, wenn die erste Liegezeit bereits abgelaufen ist.

(6) Zuständige Behörde für die Erstattung des Vermögensnachteils nach Absatz 2 und der Aufwendungen nach Absatz 4 ist die Landesdirektion Sachsen.

§ 7
Benutzungsordnung der Gemeindefriedhöfe

(1) Die Gemeinden regeln die Benutzung von Gemeindefriedhöfen und Leichenhallen sowie die Gestaltung von Grabstätten durch Satzung.

(2) [1]Den Kirchen, Religions- und Weltanschauungsgemeinschaften steht es frei, bei Bestattungen und Totengedenkfeiern nach ihren Ordnungen und Bräuchen zu verfahren. [2]Andere Feiern bedürfen einer Genehmigung des Friedhofsträgers. Die Genehmigung darf nur versagt werden, wenn Grund zu der Annahme besteht, dass durch die Art der Bestattungs- oder Totengedenkfeiern das sittliche Empfinden der Allgemeinheit oder das religiöse Empfinden der Kirchen oder der Religions- oder Weltanschauungsgemeinschaften oder ihrer Mitglieder verletzt werden könnte.

§ 8
Schließung und Aufhebung von Bestattungsplätzen

(1) [1]Bestattungsplätze können ganz oder teilweise vom Träger für weitere Erdbestattungen und Beisetzungen der Asche Verstorbener gesperrt (Schließung) oder anderen Zwecken gewidmet werden (Aufhebung). [2]Schließung und Aufhebung von Gemeindefriedhöfen sind öffentlich bekannt zu machen.

(2) [1]Die Schließung ist der für die Genehmigung nach § 1 Abs. 3 zuständigen Behörde anzuzeigen. [2]Die Träger von Friedhöfen im Sinne des § 3 Abs. 1 und von Anstaltsfriedhöfen haben die Gemeinden von der beabsichtigten Schließung zu unterrichten.

(3) [1]Bestattungsplätze dürfen nach ihrer Schließung frühestens mit Ablauf sämtlicher Ruhezeiten aufgehoben werden, sofern nicht im Einzelfall die Voraussetzungen des Absatzes 4 vorliegen. [2]Die Aufhebung bedarf der Genehmigung der nach § 1 Abs. 3 zuständigen Behörde.

(4) Die Genehmigungsbehörde kann die Schließung oder Aufhebung eines Bestattungsplatzes auch vor Ablauf der Ruhezeiten nach Anhörung des Trägers, der Gemeinde und des örtlichen Gesundheitsamtes anordnen, wenn an der Nutzung des Bestattungsplatzes zu anderen Zwecken ein zwingendes öffentliches Interesse besteht oder wenn diese Maßnahme aus Gründen der Abwehr gesundheitlicher Gefahren unumgänglich ist.

(5) [1]Bei der Aufhebung hat der Träger des Bestattungsplatzes die Leichen und die Asche Verstorbener, deren Ruhezeit noch nicht abgelaufen ist, unter Beachtung der erforderlichen Sorgfalt umzubetten und die Grabeinrichtungen zu verlegen. [2]Ein Nutzungsberechtigter, dessen Nutzungsrecht an der Grabstätte zum Zeitpunkt der Aufhebung fortbesteht, kann die Umbettung auch nach Ablauf der Ruhezeit verlangen. [3]Für Gräber im Sinne des § 6a Abs. 1 Satz 1 gilt Satz 1 entsprechend.

(6) [1]Wer die Umbettung verlangen kann, hat auch Anspruch auf Erstattung der Umbettungskosten; nach Wahl des bisherigen Nutzungsberechtigten gehören hierzu auch die Wiederherstellungskosten für die neue oder die Entschädigung für die alte Grabeinrichtung. [2]Betrifft die Aufhebung eine Wahlgrabstätte, in der weitere Bestattungen oder Beisetzungen zulässig gewesen wären, sind auch die Kosten für einen entsprechenden Wiedererwerb zu erstatten. [3]Die Ansprüche sind öffentlich-rechtlich; sie richten sich gegen die Stelle, zu deren Gunsten die Aufhebung erfolgt.

(7) [1]Die Kosten für die Umbettung eines Grabes im Sinne des § 6a Abs. 1 Satz 1 trägt der Freistaat Sachsen. [2]Zuständige Behörde für die Erstattung der Kosten ist die Landesdirektion Sachsen.

§ 8a
Gräber der Opfer von Krieg und Gewaltherrschaft

(1) Die Gemeinden sind zuständig für die Feststellung und Erhaltung der Gräber sowie die Auskunftserteilung nach § 5 des Gesetzes über die Erhaltung der Gräber der Opfer von Krieg und Gewaltherrschaft (Gräbergesetz) in der Fassung der Bekanntmachung vom 9. August 2005 (BGBl. I S. 2426), in der jeweils geltenden Fassung.

(2) Die Landesdirektion Sachsen ist zuständig für

1. die Gewährung der Ruherechtsentschädigung nach § 3 des Gräbergesetzes,

2. die Übernahme eines Grundstücks nach § 4 des Gräbergesetzes,

3. die Zustimmung zu Verlegungen nach § 6 des Gräbergesetzes und

4. die Anordnung einer Ausbettung und Identifizierung nach § 8 des Gräbergesetzes.

(3) Die vom Bund nach § 10 Gräbergesetz ausgereichten Mittel werden durch die Landesdirektion Sachsen an die Gemeinden weitergeleitet.

Zweiter Abschnitt

Leichenwesen

§ 9
Begriffsbestimmungen

(1) [1]Menschliche Leiche im Sinne des Gesetzes ist der Körper eines Menschen, der sichere Zeichen des Todes aufweist. [2]Als menschliche Leiche gilt auch ein Körperteil, ohne den ein Lebender nicht weiterleben könnte. [3]Als menschliche Leiche gilt ferner der Körper eines Neugeborenen, bei dem nach vollständigem Verlassen des Mutterleibes, unabhängig vom Durchtrennen der Nabelschnur oder von der Ausstoßung der Plazenta,

1. entweder das Herz geschlagen oder die Nabelschnur pulsiert oder die natürliche Lungenatmung eingesetzt hat (Lebendgeborenes) und das danach verstorben ist oder

2. keines der unter Nummer 1 genannten Lebenszeichen festzustellen war, das Geburtsgewicht jedoch mindestens 500 g betrug (Totgeborenes).

(2) Eine Leibesfrucht mit einem Gewicht unter 500 g, bei der nach vollständigem Verlassen des Mutterleibes keines der in Absatz 1 Nr. 1 genannten Lebenszeichen festzustellen war (Fehlgeborenes), gilt nicht als menschliche Leiche.

§ 10
Verantwortlichkeit

(1) [1]Für die Erfüllung der auf Grund dieses Gesetzes bestehenden Verpflichtungen ist der nächste voll geschäftsfähige Angehörige verantwortlich. [2]Als nächste Angehörige gelten in der Reihenfolge der Aufzählung

1. der Ehegatte oder der Lebenspartner nach dem Gesetz über die Eingetragene Lebenspartnerschaft (Lebenspartnerschaftsgesetz – LPartG) vom 16. Februar 2001 (BGBl. I S. 266), zuletzt geändert durch Artikel 2 des Gesetzes vom 21. Dezember 2007 (BGBl. I S. 3189, 3191), in der jeweils geltenden Fassung,

2. die Kinder,

3. die Eltern,

4. die Geschwister,

5. der Partner einer auf Dauer angelegten nichtehelichen Lebensgemeinschaft nach § 7 Abs. 3 Nr. 3 in Verbindung mit Abs. 3a des Zweiten Buches Sozialgesetzbuch (SGB II) – Grundsicherung für Arbeitsuchende – (Artikel 1 des Gesetzes vom 24. Dezember 2003, BGBl. I S. 2954, 2955), das zuletzt durch Artikel 8 und 9 des Gesetzes vom 2. März 2009 (BGBl. I S. 416, 429, 430) geändert worden ist, in der jeweils geltenden Fassung,

6. der sonstige Sorgeberechtigte,

7. die Großeltern,

8. die Enkelkinder,

9. sonstige Verwandte bis zum 3. Grade.

[3]Kommt für die Verantwortlichkeit ein Paar (Nummern 3 und 7) oder eine Mehrheit von Personen (Nummern 2, 4, 8 und 9) in Betracht, so geht jeweils die ältere Person der jüngeren in der Verantwortlichkeit vor, es sei denn, die Verantwortlichen haben einvernehmlich eine andere Lösung getroffen.

(2) Hat ein Bestattungsunternehmer oder ein Dritter durch Vertrag mit dem Verstorbenen zu dessen Lebzeiten Verpflichtungen, die nach diesem Gesetz bestehen, übernommen, so gilt der Bestattungsunternehmer oder der Dritte hinsichtlich dieser Verpflichtungen als verantwortlich.

(3) Ist ein Bestattungspflichtiger im Sinne des Absatzes 1 und 2 nicht vorhanden oder nicht rechtzeitig zu ermitteln oder kommt er seiner Pflicht nicht nach und veranlasst kein anderer die Bestattung, hat die für den Sterbeort zuständige Ortspolizeibehörde auf Kosten des Bestattungspflichtigen für die Bestattung zu sorgen. Abweichend von Absatz 1 Satz 3 haften ein Paar oder eine Mehrheit von Personen der Ortspolizeibehörde als Gesamtschuldner für die Bestattungskosten. Diese werden durch Leistungsbescheid festgesetzt. Widerspruch und Anfechtungsklage haben keine aufschiebende Wirkung.

§ 11
Pflicht zur Veranlassung der Leichenschau; Benachrichtungspflichten

(1) [1]Der nach § 10 Abs. 1 Verantwortliche hat nach dem Sterbefall unverzüglich die Leichenschau zu veranlassen. [2]Bei Sterbefällen

1. in Krankenhäusern, Altenheimen oder anderen Gemeinschaftseinrichtungen oder

2. in Betrieben, öffentlichen Einrichtungen, Verkehrsmitteln oder während einer Veranstaltung

obliegt die Pflicht zur Veranlassung der Leichenschau vorrangig dem Leiter der Einrichtung oder des Betriebes, dem Fahrzeugführer oder dem Veranstalter.

(2) [1]Wer eine menschliche Leiche auffindet oder wer beim Eintritt des Todes eines Menschen anwesend ist, hat unverzüglich eine der in § 10 Abs. 1 genannten Personen oder eine Polizeidienststelle zu benachrichtigen. [2]Wer eine tote Leibesfrucht im Sinne des § 9 Abs. 2 auffindet, hat unverzüglich die nächste Polizeidienststelle zu unterrichten.

(3) Ist nur eine Polizeidienststelle benachrichtigt oder kommt keiner der in Absatz 1 bezeichneten Verantwortlichen seiner Pflicht zur Veranlassung der Leichenschau nach, wird die Leichenschau von der Polizeidienststelle veranlasst.

(4) [1]Die Kosten der Leichenschau und der Ausstellung der Todesbescheinigung sind von demjenigen zu tragen, der für die Kosten der Bestattung aufzukommen hat. [2]Dessen Recht, aufgrund anderer gesetzlicher Vorschrift oder aufgrund besonderer vertraglicher Vereinbarung die Erstattung der Kosten von Dritten zu verlangen, bleibt unberührt.

§ 12
Ärztliche Leichenschaupflicht

(1) Jede menschliche Leiche ist zur Feststellung des Todes, des Todeszeitpunkts, der Todesart und der Todesursache von einem Arzt zu untersuchen (Leichenschau).

(2) [1]Zur Vornahme der Leichenschau sind verpflichtet:

1. jeder erreichbare, in der ambulanten Versorgung tätige Arzt, vorrangig jedoch der behandelnde Hausarzt im Rahmen seines Sicherstellungsauftrages,
2. die während des Kassenärztlichen Bereitschaftsdienstes tätigen Ärzte,
3. bei Sterbefällen in Krankenhäusern oder vergleichbaren Einrichtungen jeder dort tätige Arzt, der von der Leitung des Krankenhauses oder der Einrichtung dazu bestimmt ist,
4. bei Sterbefällen in einem Fahrzeug des Rettungsdienstes oder eines sonstigen organisierten Krankentransportwesens der in dem jeweils nächstgelegenen Krankenhaus diensthabende Arzt.

[2]Die Leichenschau kann auch von einem Facharzt für Rechtsmedizin übernommen werden, wenn dieser sich bereit erklärt hat, die Leichenschau anstelle des nach Satz 1 oder Absatz 4 verpflichteten Arztes durchzuführen. [3]Der nach § 10 Abs. 1 Verantwortliche ist berechtigt, den Arzt, der den Verstorbenen wegen der dem Tode unmittelbar vorausgegangenen Krankheit behandelt hat, als Leichenschauarzt abzulehnen. [4]Macht der Angehörige von diesem Recht Gebrauch, hat er unverzüglich selbst zu veranlassen, dass ein anderer Arzt die Leichenschau vornimmt.

(3) [1]Ärzte, die sich im Rettungsdiensteinsatz befinden, können sich auf die Feststellung des Todes und auf seine Dokumentation in einer amtlichen vorläufigen Todesbescheinigung nach dem diesem Gesetz als Anlage 2 beigefügten Muster beschränken. [2]Zu einer umfassenden Leichenschau sind diese Ärzte nicht verpflichtet. [3]Liegt kein weiterer Rettungsdiensteinsatz aktuell vor, soll der Arzt die vollständige Leichenschau durchführen. [4]Das Rettungsdienstprotokoll ist bei der Leiche zurückzulassen. [5]Das Rettungsdienstprotokoll ist vom Leichenschauarzt zusammen mit dem Blatt 3 des vertraulichen Teils der Todesbescheinigung zu verschließen und verbleibt bei der Leiche. [6]Beschränkt sich ein im Rettungsdiensteinsatz befindlicher Arzt auf die vorläufige Todesbescheinigung und sorgt er nicht selbst dafür, dass ein anderer

Arzt die vollständige Leichenschau durchführt, hat dies der nach § 11 Abs. 1 Satz 2 oder der nach § 10 Verantwortliche zu veranlassen.

(4) Ist ein zur Leichenschau verpflichteter Arzt im Einzelfall aus wichtigem Grund an der Durchführung der Leichenschau verhindert, hat er unverzüglich eine Vertretung zu bestellen.

§ 13
Durchführung der äußeren Leichenschau

(1) [1]Die Leichenschau soll an dem Ort, an dem der Tod eingetreten oder die Leiche aufgefunden worden ist, unverzüglich vorgenommen werden. [2]Der Arzt und die von ihm hinzugezogenen Sachverständigen und Gehilfen sind berechtigt, jederzeit den Ort zu betreten, an dem sich die Leiche befindet. [3]Der Inhaber der tatsächlichen Gewalt hat ihnen Grundstücke, Räume und, soweit erforderlich, auch bewegliche Sachen zugänglich zu machen. [4]Das Grundrecht der Unverletzlichkeit der Wohnung (Artikel 13 Abs. 1 des Grundgesetzes, Artikel 30 Abs. 1 der Verfassung des Freistaates Sachsen) wird eingeschränkt. [5]Befindet sich die Leiche nicht in einem geschlossenen Raum oder ist aus anderen Gründen eine vollständige Leichenschau nicht möglich oder nicht zweckmäßig, kann der Arzt zunächst entsprechend § 12 Abs. 3 Satz 1 verfahren; er hat alsdann die Leichenschau an einem hierfür besser geeigneten Ort fortzusetzen und die vollständige Todesbescheinigung auszustellen.

(2) [1]Angehörige, Hausbewohner und Nachbarn sowie Personen, die den Verstorbenen während einer dem Tode vorausgegangenen Krankheit behandelt oder gepflegt haben, sind verpflichtet, dem Arzt auf Verlangen Auskunft über die Krankheit oder andere Gesundheitsschädigungen des Verstorbenen oder über sonstige für seinen Tod möglicherweise ursächliche Ereignisse zu erteilen. [2]Sie können die Auskunft auf solche Fragen verweigern, deren Beantwortung sie selbst oder einen ihrer in § 52 Abs. 1 Nr. 1 bis 3 der Strafprozessordnung bezeichneten Angehörigen der Gefahr strafrechtlicher Verfolgung oder eines Verfahrens nach dem Gesetz über Ordnungswidrigkeiten aussetzen würde.

(3) [1]Die Leiche ist zu entkleiden und durch den Arzt unter Einbeziehung aller Körperregionen, insbesondere auch des Rückens, der Hals- und Nackenregion und der Kopfhaut, gründlich zu untersuchen. [2]Der Arzt hat hierbei vor allem auf Merkmale und Zeichen zu achten, die auf einen nichtnatürlichen Tod hindeuten. [3]Als nichtnatürlich ist ein Tod anzunehmen, der durch Selbsttötung, durch Komplikationen medizinischer Behandlungen, durch einen Unfall oder durch eine äußere Einwirkung, bei der ein Verhalten eines Dritten ursächlich gewesen sein könnte (Tod durch fremde Hand), eingetreten ist. [4]Stellt der Arzt bereits vor einer Leichenschau oder vor einer näheren Untersuchung der Leiche Anhaltspunkte für einen nichtnatürlichen Tod fest oder

handelt es sich um die Leiche eines Unbekannten, hat er von einer Entkleidung der Leiche abzusehen und unverzüglich die zuständige Polizeidienststelle zu verständigen. [5]Der Arzt hat dafür zu sorgen, dass bis zum Eintreffen der Polizeibeamten an der Leiche und deren Umgebung keine Veränderungen vorgenommen werden. [6]Er hat in gleicher Weise zu verfahren, wenn sich Anhaltspunkte für einen nichtnatürlichen Tod erst nach der Entkleidung der Leiche oder im Verlauf ihrer näheren Untersuchung ergeben.

(4) Ergibt die Untersuchung der Leiche keine Anhaltspunkte für einen nichtnatürlichen Tod, legen aber die Gesamtumstände Zweifel an einem natürlichen Tod nahe, muss die Todesart als ungeklärt in der Todesbescheinigung vermerkt und die Polizei benachrichtigt werden.

(5) Hatte der Verstorbene an einer meldepflichtigen Krankheit im Sinne des § 6 des Gesetzes zur Verhütung und Bekämpfung von Infektionskrankheiten beim Menschen (Infektionsschutzgesetz – IfSG) vom 20. Juli 2000 (BGBl. I S. 1045), das zuletzt durch Artikel 2 des Gesetzes vom 13. Dezember 2007 (BGBl. I S. 2904, 2915) geändert worden ist, in der jeweils geltenden Fassung, gelitten oder besteht ein solcher Verdacht und ist zu befürchten, dass die Erreger dieser Krankheit durch den Umgang mit der Leiche verbreitet werden (Ansteckungsgefahr), hat der Arzt unverzüglich das Gesundheitsamt zu benachrichtigen und dafür zu sorgen, dass die Leiche, der Sarg und der Umschlag der Todesbescheinigung entsprechend gekennzeichnet werden.

(6) Weist die Leiche Zeichen radioaktiver Stoffe auf oder wird dies aufgrund einer radioaktiven Behandlung vermutet, so hat der Arzt dies auf der Todesbescheinigung und auf dem Sarg zu vermerken.

§ 14
Todesbescheinigung

(1) [1]Nach Beendigung der Leichenschau ist unverzüglich eine Todesbescheinigung nach dem diesem Gesetz als Anlage 1 beigefügten Muster sorgfältig auszustellen. [2]Die Todesbescheinigung enthält einen nichtvertraulichen und einen aus Blatt 1 bis 4 bestehenden vertraulichen Teil.

(2) [1]Die Todesbescheinigung muss über die verstorbene Person die folgenden Angaben enthalten:

1. Name, Geschlecht,

2. letzte Wohnung,

3. minutengenauer Zeitpunkt des Todes, Ort des Todes oder Auffindens, bei Totgeborenen außerdem das Geburtsgewicht; ein Sterbezeitraum darf nur angegeben werden, wenn der minutengenaue Todeszeitpunkt nicht bekannt ist,

4. Name, Anschrift und Telefonnummer des Arztes, der die verstorbene Person zuletzt behandelt hat, oder Angabe des Krankenhauses, in dem die verstorbene Person zuletzt behandelt wurde,

5. Angaben über übertragbare Krankheiten oder radioaktive Verstrahlung,

6. Art des Todes (natürlicher, nichtnatürlicher oder unaufgeklärter Tod),

7. Angaben zur Krankheitsanamnese,

8. unmittelbare oder mittelbare Todesursachen sowie weitere wesentliche Krankheiten oder Veränderungen zur Zeit des Todes mit der vollständigen Textangabe und der Verschlüsselung nach der Internationalen Klassifikation der Krankheiten ICD-10-WHO in der jeweils aktuellen Version,

9. Angaben über durchgeführte Reanimationsbehandlungen,

10. bei Verdacht eines nichtnatürlichen Todes: Angaben über die Art des nichtnatürlichen Todes (§ 13 Abs. 3 Satz 3),

11. bei Frauen: Angaben darüber, ob Anzeichen dafür vorliegen, dass in den letzten 3 Monaten eine Schwangerschaft bestand,

12. bei Totgeborenen und bei Kindern unter einem Jahr: Angaben über die Stätte der Geburt, über Körpergewicht und -länge bei der Geburt, über das Vorliegen einer Mehrlingsgeburt und über Erkrankungen der Mutter während der Schwangerschaft; bei Kindern, die innerhalb der ersten 24 Stunden nach der Geburt gestorben sind, Angabe der Anzahl der Lebensstunden.

[2]Die in den Nummern 7 bis 12 bezeichneten Angaben dürfen nur in dem verschließbaren, von außen nicht lesbaren vertraulichen Teil der Todesbescheinigung enthalten sein.

(3) [1]Blatt 4 des vertraulichen Teils der Todesbescheinigung ist für den Leichenschauarzt bestimmt und kann von ihm entnommen werden. [2]Blatt 3 des vertraulichen Teils, das entsprechend zu kennzeichnen und das zu verschließen ist, verbleibt bei der Leiche. Blatt 1 und 2 des vertraulichen Teils sind von dem Leichenschauarzt zu verschließen und zusammen mit dem nichtvertraulichen Teil derjenigen Person auszuhändigen, die nach dem Personenstandsgesetz zur Anzeige des Todes beim Standesamt verpflichtet ist. [3]Diese oder der von ihr beauftragte Bestattungsunternehmer hat diese Exemplare der Todesbescheinigung spätestens am 3. auf den Tod folgenden Werktag dem Standesamt vorzulegen; der Samstag gilt nicht als Werktag im Sinne dieser Regelung. [4]Der Standesbeamte öffnet Blatt 1 und 2 des vertraulichen Teils der Todesbescheinigung, beurkundet den Sterbefall im Sterbebuch, die Totgeburt im Geburtenbuch, vermerkt die Beurkundung in der Todesbescheinigung, behält den nichtvertraulichen Teil der Todesbescheinigung in seinen Unterlagen und leitet spätestens am nächsten Werktag Blatt 1 und 2 des vertraulichen Teils an das Gesundheitsamt des Sterbeortes weiter.

(4) [1]Aus Gründen der Rechtssicherheit, der Gefahrenabwehr und zu statistischen Zwecken überprüft das Gesundheitsamt des Sterbeortes den Inhalt des vertraulichen Teils der Todesbescheinigung und gegebenenfalls des Obduktionsscheins auf Vollständigkeit und Schlüssigkeit der von dem Arzt nach der Leichenschau oder der Obduktion vorgenommenen Eintragungen. [2]Ärzte, die die äußere oder die innere Leichenschau durchgeführt haben, sind verpflichtet, die zur Überprüfung und Vervollständigung der Todesbescheinigung oder des Obduktionsscheins erforderlichen Auskünfte zu erteilen. [3]Ärzte und sonstige Personen, die den Verstorbenen zuletzt behandelt oder gepflegt haben, sind auf Aufforderung der jeweils zuständigen Behörde zu näherer Auskunft verpflichtet. [4]Soweit sie über Krankenunterlagen verfügen, sind sie auf Verlangen auch zu deren Vorlage verpflichtet. [5]Eine Verweigerung der Auskunft nach Satz 2 und 3 oder eine Verweigerung der Vorlage der Krankenunterlagen ist nur zulässig, wenn sich der Arzt selbst oder einen seiner in § 52 Abs. 1 Nr. 1 bis 3 der Strafprozessordnung bezeichneten Angehörigen der Gefahr strafrechtlicher Verfolgung oder eines Verfahrens nach dem Gesetz über Ordnungswidrigkeiten aussetzen würde.

(5) Das Gesundheitsamt des Sterbeortes leitet Blatt 2 des vertraulichen Teils an das Statistische Landesamt weiter.

(6) Die Daten der Todesbescheinigung können auch elektronisch übermittelt werden.

(7) [1]Das Gesundheitsamt des Sterbeortes bewahrt die Todesbescheinigung und die ihm von auswärtigen Stellen zugesandten gleichartigen Bescheinigungen 30 Jahre lang auf. [2]Es übermittelt dem Gesundheitsamt des letzten Hauptwohnortes eine Kopie der Todesbescheinigung. [3]Auf Antrag können die Gesundheitsämter Einsicht in die Todesbescheinigung gewähren oder Auskünfte daraus erteilen, wenn

1.der Antragsteller ein berechtigtes Interesse an der Kenntnis der Todesumstände einer namentlich bezeichneten verstorbenen Person glaubhaft macht und kein Grund zu der Annahme besteht, dass durch die Offenbarung schutzwürdige Belange des Verstorbenen oder seiner Hinterbliebenen beeinträchtigt werden oder

2. Hochschulen oder andere mit wissenschaftlicher Forschung befasste Stellen die Angaben für ein wissenschaftliches Vorhaben benötigen und wenn dem wissenschaftlichen Interesse an der Durchführung des Forschungsvorhabens größeres Gewicht als den Belangen des Verstorbenen oder seiner Hinterbliebenen beizumessen ist. § 36 Abs. 2 bis 6 des Gesetzes zum Schutz der informationellen Selbstbestimmungen im Freistaat Sachsen (Sächsisches Datenschutzgesetz – SächsDSG) vom 25. August 2003 (SächsGVBl. S. 330), in der jeweils geltenden Fassung, ist entsprechend anzuwenden.

(8) [1]Absatz 7 ist auch auf die vor Inkrafttreten dieses Gesetzes erstellten Totenscheine anzuwenden. [2]Die Aufbewahrungsfrist beginnt in diesen Fällen mit dem Zugang der Totenscheine bei dem örtlich zuständigen Gesundheitsamt.

§ 15
Innere Leichenschau

(1) [1]Eine innere Leichenschau (Obduktion) ist zulässig, wenn sie

1. von einem Richter oder Staatsanwalt oder der nach § 26 Abs. 3 IfSG zuständigen Behörde angeordnet ist,

2. zur Durchsetzung berechtigter Interessen der Hinterbliebenen, insbesondere zur Feststellung versicherungsrechtlicher Leistungsansprüche, erforderlich ist,

3. der Klärung des Verdachts dient, dass der Tod durch einen medizinischen Behandlungsfehler verursacht sein könnte, und sofern der nach § 10 Abs. 1 verantwortliche Angehörige sie wünscht,

4. durch ein beachtliches Interesse an der Überprüfung der vorherigen Diagnose oder durch ein gewichtiges medizinisches Forschungsinteresse gerechtfertigt ist, sofern ihr entweder der Verstorbene zu Lebzeiten zugestimmt hat, oder, sofern von ihm eine Erklärung hierzu nicht vorliegt, der nach § 10 Abs. 1 verantwortliche Angehörige zustimmt oder

5. von dem zuständigen Gesundheitsamt bei einem plötzlich und unerwartet eingetretenen Todesfall, an dessen Aufklärung ein besonderes Interesse besteht, angeordnet wird. Ein besonderes öffentliches Interesse an der Obduktion ist insbesondere dann anzunehmen, wenn wegen Unklarheit der Todesursache, zur Beweissicherung oder zur Qualitätssicherung die Obduktion als so gewichtig anzusehen ist, dass sie auch ohne Zustimmung nach Nummer 4 durchzuführen ist. Der nach § 10 Abs. 1 verantwortliche Angehörige soll zuvor gehört werden.

[2]In den Fällen der Nummern 4 und 5 ist das entsprechende Feld auf der Todesbescheinigung zu kennzeichnen.

(2) [1]Mit der inneren Leichenschau sollen nur Fachärzte für Pathologie oder für Rechtsmedizin betraut werden. [2]Dem Arzt sind die Krankenunterlagen zur Verfügung zu stellen. [3]Die Obduktion ist unter Wahrung der Ehrfurcht vor dem toten Menschen durchzuführen und auf das zur Erreichung ihres Zwecks notwendige Maß, in der Regel auf die Öffnung der 3 Körperhöhlen, zu beschränken. [4]Gewebeproben dürfen entnommen werden, soweit der Zweck der Obduktion dies erfordert. [5]Für die Durchführung der von einem Staatsanwalt oder einem Richter angeordneten Leichenöffnung bleiben die Vorschriften der §§ 87 bis 91 der Strafprozessordnung unberührt.

(3) [1]Teilsektionen, die der Entfernung nicht verweslicher oder nicht brennbarer Implantate, insbesondere von Metallendoprothesen, dienen, sind auch

zulässig, wenn die Voraussetzungen des Absatzes 1 nicht vorliegen. [2]Auf sie ist Absatz 2 Satz 1 nicht anzuwenden.

(4) [1]Ergeben sich erst während der Leichenöffnung Anhaltspunkte für einen nichtnatürlichen Tod, ist § 13 Abs. 3 Satz 4 und 5 entsprechend anzuwenden. [2]Die Leichenöffnung darf in diesem Fall nur mit Zustimmung der zuständigen Polizeidienststelle fortgesetzt werden.

(5) Über die Obduktion hat der Arzt, der sie durchführt, unverzüglich nach Abschluss auch aller eventuell notwendigen Zusatzuntersuchungen einen Obduktionsschein nach dem diesem Gesetz als Anlage 3 beigefügten Muster sorgfältig auszustellen.

(6) Nach dem Vorliegen aller Untersuchungsergebnisse ist der vervollständigte Obduktionsschein dem Gesundheitsamt des Sterbeortes zu übersenden.

(7) [1]Soweit die Kostenpflicht nicht in anderen Gesetzen besonders geregelt ist, sind die Kosten der inneren Leichenschau von demjenigen zu tragen, der ihre Vornahme veranlasst hat oder in dessen Interesse sie erfolgt. [2]Dessen Recht, aufgrund anderer gesetzlicher Vorschrift oder aufgrund besonderer vertraglicher Vereinbarung die Erstattung der Kosten von Dritten zu verlangen, bleibt unberührt.

§ 16
Einsargung und Überführung

(1) [1]Leichen sind nach Abschluss der Leichenschau unverzüglich einzusargen und, sofern die zuständige Behörde im Einzelfall nicht eine Ausnahme zulässt, unverzüglich in eine Leichenhalle oder in einen Raum zu überführen, der ausschließlich der Aufbewahrung von Leichen dient. [2]Dies gilt nicht, wenn die Leiche zur Durchführung einer inneren Leichenschau oder im Zusammenhang mit anderen ärztlichen Maßnahmen oder wissenschaftlichen Untersuchungen in eine andere Einrichtung überführt werden soll. [3]Vor der Überführung und während oder in unmittelbarem Zusammenhang mit der Bestattungsfeier kann der Tote offen aufgebahrt werden. [4]Außer im Falle des Satzes 2 muss die Überführung spätestens 24 Stunden nach Feststellung des Todes beginnen.

(2) Ist der Todesfall in einem Krankenhaus, einem Alten- oder Pflegeheim eingetreten, soll den Angehörigen vor der Überführung die Möglichkeit gegeben werden, in würdiger Weise Abschied zu nehmen.

(3) Die Leiche muss in einem festen, gut abgedichteten und aus umweltgerecht abbaubarem Material bestehenden Sarg gelegt werden, dessen Boden grundsätzlich mit einer 5 bis 10 cm hohen Schicht aus Sägemehl, Sägespänen, Holzwolle oder anderen geeigneten aufsaugenden Stoffen bedeckt ist.

(4) [1]Hatte der Verstorbene an einer meldepflichtigen Krankheit im Sinne des § 6 IfSG gelitten oder besteht ein solcher Verdacht und geht von der Leiche eine Ansteckungsgefahr aus, hat der behandelnde oder sonst hinzugezogene Arzt unverzüglich das Gesundheitsamt zu unterrichten, sofern dies nicht gemäß § 13 Abs. 5 bereits der Leichenschauarzt getan hat. [2]Den Anweisungen des Gesundheitsamtes ist Folge zu leisten. [3]Soweit das Gesundheitsamt im Einzelfall keine andere Anweisung gibt, ist die Leiche unverzüglich einzusargen. [4]Der Sarg ist sofort zu schließen und entsprechend zu kennzeichnen.

(5) [1]Leichenhallen und sonstige zur Aufbahrung von Leichen dienende Räume müssen gut lüftbar, kühl, leicht zu reinigen sowie gegen das Betreten Unbefugter und das Eindringen von Tieren geschützt sein. [2]Räume zur ausschließlichen Aufbewahrung von Leichen dürfen darüber hinaus eine Raumtemperatur von maximal 8 Grad Celsius aufweisen. [3]Leichenhallen müssen darüber hinaus über einen Wasseranschluß und einen Wasserauslauf verfügen. [4]Die Räumlichkeiten unterliegen in hygienischer Hinsicht der Aufsicht des Gesundheitsamtes.

§ 17
Beförderung von Leichen

(1) [1]Zur Beförderung von Leichen im Straßenverkehr dürfen nur Fahrzeuge benutzt werden, die zur Leichenbeförderung eingerichtet sind und die den Mindestanforderungen genügen, die nach den anerkannten Regeln der Technik an sie zu stellen sind (Leichenwagen). [2]Die Beförderung von Leichen in Kraftfahrzeuganhängern, die nicht als Leichenwagen anzusehen sind, ist nicht zulässig. [3]Die Sätze 1 und 2 gelten nicht für die Bergung von Leichen, insbesondere nicht für die Beförderung tödlich Verunglückter von der Unfallstelle.

(2) Das Gesundheitsamt kann Ausnahmen von Absatz 1 Satz 1 und 2 zulassen.

(3) [1]Für die Beförderung von Leichen, die ins Ausland oder in ein anderes Land der Bundesrepublik Deutschland, das durch Gesetz oder Verordnung einen Leichenpaß vorschreibt, überführt werden sollen, stellt das Gesundheitsamt des Sterbeortes einen Leichenpaß aus. [2]§ 18b Abs. 2 bis 5 bleibt unberührt. [3]Für den Leichenpaß ist das diesem Gesetz als Anlage 4 beigefügte Muster zu verwenden.

(4) [1]Bei der Beförderung von Leichen aus dem Ausland hat der Beförderer einen Leichenpaß oder ein vergleichbares Dokument mitzuführen, das nach den für den Herkunftsort geltenden Vorschriften ausgestellt ist. [2]Bei Beförderungen von Leichen aus einem anderen Land der Bundesrepublik Deutschland genügt eine nach den Vorschriften dieses Landes ausgestellte Bescheinigung, aus der sich die Zulässigkeit der Beförderung ergibt. [3]Ist eine Leiche

ohne den Paß oder ein vergleichbares Dokument nach Satz 1 oder ohne die Bescheinigung nach Satz 2 in den Freistaat Sachsen befördert worden, ist die weitere Beförderung zu dem bestimmungsgemäßen Bestattungsort gleichwohl zuzulassen. [4]§ 18a Abs. 3 und § 18b Abs. 4 bleiben unberührt.

(5) [1]Bei der Beförderung einer Leiche über die Grenze der Bundesrepublik Deutschland ins Ausland muss der Sarg undurchlässig und mit saugfähigen Stoffen ausgekleidet sein. [2]Der Sarg muss

1. entweder aus einem äußeren Holzsarg mit einer Wandstärke von mindestens 20 mm und einem sorgfältig verlöteten inneren Sarg aus Zink oder aus einem anderen Stoff, der sich selbst zersetzt, oder

2. aus einem einzigen sorgfältig abgedichteten Holzsarg mit einer Wandstärke von mindestens 30 mm, der mit einer Schicht aus Zink oder aus einem anderen Stoff, der sich selbst zersetzt, ausgekleidet ist,

bestehen.

(6) Bei der Beförderung einer Leiche auf dem Luftweg ist der Sarg mit einer geeigneten Druckausgleichsvorrichtung zu versehen.

(7) [1]Bei der Beförderung oder dem Versand einer Urne mit der Asche eines Verstorbenen genügt es anstelle der in den Absätzen 1 bis 6 geregelten Anforderungen, wenn die Urne sicher verschlossen mit den Identitätsdaten des Toten gekennzeichnet und ihr der Einäscherungsschein sowie der Urnenaufnahmeschein des Friedhofs, der zur Aufnahme der Asche bestimmt ist, beigefügt sind. [2]Soll die Urne auf Wunsch des Verstorbenen von einem Schiff aus auf hoher See beigesetzt werden, genügt anstelle des Urnenaufnahmescheins nach Satz 1 die Genehmigung der für die Seebestattung zuständigen Behörde des Küstenlandes.

Dritter Abschnitt

Bestattungswesen

§ 18
Allgemeine Vorschriften zur Bestattung

(1) [1]Jede menschliche Leiche muss bestattet werden. [2]Die Bestattung im Freistaat Sachsen ist nur auf einem in § 1 Abs. 1 bezeichneten Bestattungsplatz zulässig.

(2) [1]Auf Wunsch eines Elternteils sind auch Fehlgeborene (§ 9 Abs. 2) zur Bestattung zuzulassen. [2]Zum Nachweis einer solchen Fehlgeburt ist dem Friedhofsträger eine formlose ärztliche Bestätigung vorzulegen.

(3) [1]Für Ort, Art und Durchführung der Bestattung ist der Wille des Verstorbenen maßgebend, soweit gesetzliche Bestimmungen oder zwingende öffentliche Belange nicht entgegenstehen. [2]Bei Verstorbenen, deren Wille nicht bekannt ist, und bei Verstorbenen, die das 14. Lebensjahr nicht vollendet hatten oder die geschäftsunfähig waren, ist der Wille des nach § 10 Abs. 1 Verantwortlichen maßgebend. [3]Für Verstorbene ohne Hinterbliebene ist die ortsübliche Bestattungsart zu wählen. [4]Bei der Vorbereitung und Durchführung der Bestattung sind die Würde und die Religionszugehörigkeit des Verstorbenen und das sittliche Empfinden der Allgemeinheit zu achten.

(4) [1]Die Bestattung kann als Erd- oder Feuerbestattung vorgenommen werden. [2]Implantate sind vor der Erd- oder Feuerbestattung vom Leichenschauarzt oder Bestatter zu entfernen, wenn sonst Schäden für die Umwelt oder an der Verbrennungsanlage zu befürchten wären.

(5) Vor einer Beisetzung ist dem Friedhofsträger die Sterbeurkunde im Original vorzulegen.

(6) [1]Sofern Fehlgeborene (§ 9 Abs. 2) und Feten aus operativen und medikamentösen Schwangerschaftsabbrüchen nicht gemäß Absatz 2 bestattet werden, sind sie innerhalb eines Jahres zu bestatten, sofern sie nicht zu medizinischen, pharmazeutischen oder wissenschaftlichen Zwecken verwendet oder sofern sie nicht als Beweismittel aufbewahrt werden. [2]Die Bestattung kann auch gemeinschaftlich oder anonym erfolgen.

(7) Abgetrennte Körperteile von Lebenden und Teile von Leichen einschließlich der Teile von Leichen von unmittelbar vor oder nach der Geburt verstorbenen Kindern sind von dem Inhaber des Gewahrsams innerhalb eines Jahres hygienisch einwandfrei zu beseitigen, sofern diese Teile nicht zum Zwecke der Übertragung auf Menschen entnommen worden sind und für diesen Zweck verwendet werden (Transplantate) oder sofern sie nicht zu medizinischen, pharmazeutischen oder wissenschaftlichen Zwecken verwendet oder sofern sie nicht als Beweismittel aufbewahrt werden.

(8) Bei einem Körperspender für die Anatomie muss vor der anatomischen Verwendung der Leiche die nach § 18b Abs. 2 vorgeschriebene besondere amtliche Leichenschau (2. Leichenschau) durchgeführt werden. 17

§ 18a
Erdbestattung

(1) Erdbestattung ist die Beisetzung der Leiche in einer Grabstätte.

(2) Die Erdbestattung ist zulässig, wenn der Standesbeamte des Sterbeortes auf der Todesbescheinigung vermerkt hat, dass der Sterbefall in das Sterbebuch, die Totgeburt in das Geburtenbuch eingetragen ist.

(3) [1]Bei nichtnatürlichen Todesfällen oder bei der Leiche eines Unbekannten ist zusätzlich das schriftliche Einverständnis der Staatsanwaltschaft oder des Ermittlungsrichters beim Amtsgericht des Sterbeortes erforderlich. [2]Dies gilt nicht bei einem medizinisch indizierten Schwangerschaftsabbruch.

§ 18b
Feuerbestattung

(1) Feuerbestattung ist die Einäscherung der Leiche und die Beisetzung ihrer Asche in einer Grabstätte.

(2) [1]Eine Einäscherung ist zulässig, wenn der Standesbeamte des Sterbeortes auf der Todesbescheinigung vermerkt hat, dass der Sterbefall in das Sterbebuch, die Totgeburt in das Geburtenbuch eingetragen ist. [2]Zusätzlich muss eine Unbedenklichkeitserklärung des Gesundheitsamtes des Einäscherungsortes vorliegen, aus der hervorgeht, dass aufgrund einer zweiten, von einem Facharzt für Rechtsmedizin durchgeführten Leichenschau keine Bedenken gegen eine Einäscherung bestehen. [3]Falls in einer Region nicht genügend Fachärzte für Rechtsmedizin für die Durchführung der zweiten Leichenschau zur Verfügung stehen, kann diese Leichenschau auch von einem in der Leichenschau erfahrenen Facharzt für Pathologie durchgeführt werden. [4]Eine Kopie der Unbedenklichkeitserklärung ist dem Gesundheitsamt des Sterbeortes zuzuleiten. [5]Die Sätze 1 und 2 gelten nicht bei einem medizinisch indizierten Schwangerschaftsabbruch.

(3) Die zweite Leichenschau entfällt, wenn bereits die ärztliche Leichenschau von einem Facharzt für Rechtsmedizin vorgenommen wurde.

(4) Ergeben sich bei der zweiten Leichenschau Anhaltspunkte für einen nichtnatürlichen Tod oder handelt es sich um die Leiche eines Unbekannten, ist anstelle der Unbedenklichkeitserklärung des Gesundheitsamtes das schriftliche Einverständnis der Staatsanwaltschaft oder des Ermittlungsrichters beim Amtsgericht des Sterbeortes erforderlich.

(5) [1]Wird die Leiche zur Einäscherung in ein anderes Land der Bundesrepublik Deutschland überführt, hat das Gesundheitsamt des Sterbeortes eine zweite Leichenschau zu veranlassen, es sei denn, in dem jeweiligen Bundesland ist ebenfalls eine zweite Leichenschau vorgeschrieben. [2]Bei Überführung einer Leiche zur Einäscherung in das Ausland ist immer eine zweite Leichenschau durchzuführen. [3]Absatz 3 bleibt unberührt.

(6) Die Urne zur Beisetzung der Asche eines Verstorbenen muss innerhalb der Ruhefrist umweltgerecht abbaubar sein.

§ 19
Fristen für die Bestattung

(1) [1]Die Erdbestattung oder Einäscherung darf frühestens 48 Stunden nach Feststellung des Todes erfolgen. [2]Sie muss innerhalb von 8 Tagen nach Feststellung des Todes durchgeführt werden. [3]Samstage, Sonntage und Feiertage werden bei der Fristberechung nicht mitgezählt. [4]Satz 1 gilt nicht für Leichen, die zu medizinischen oder wissenschaftlichen Zwecken in ein Krankenhaus oder eine wissenschaftliche Einrichtung gebracht werden. [5]Diese Leichen sind zu bestatten, sobald sie nicht mehr diesen Zwecken dienen.

(2) Die Asche eines Verstorbenen ist innerhalb von 6 Monaten nach der Einäscherung auf einem Bestattungsplatz (§ 1 Abs. 1) beizusetzen.

(3) Das Gesundheitsamt des Sterbeortes kann die 48-Stunden-Frist verkürzen, wenn andernfalls gesundheitliche oder hygienische Gefahren zu befürchten wären; es kann die 8-Tage-Frist verlängern, wenn gesundheitliche oder hygienische Bedenken nicht entgegenstehen.

(4) § 18a Abs. 2 und 3, § 18b Abs. 2 und 4 dieses Gesetzes sowie § 28 des Personenstandsgesetzes vom 19. Februar 2007 (BGBl. I S. 122), das zuletzt durch Artikel 12 des Gesetzes vom 17. Dezember 2008 (BGBl. I S. 2586) geändert worden ist, und § 159 Abs. 2 StPO bleiben unberührt.

§ 20
Einäscherungsanlagen

(1) [1]Leichen dürfen nur in Einäscherungsanlagen eingeäschert werden, deren Betrieb den immissionsschutzrechtlichen Bestimmungen entspricht. [2]Eine Einäscherungsanlage muss mit einer Leichenhalle und mit einem Raum zur Durchführung der äußeren Leichenschau verbunden sein. [3]Ein Raum zur Durchführung der inneren Leichenschau sollte zur Verfügung stehen. 4Einäscherungen dürfen nur in einem hierfür geeigneten umweltverträglichen Sarg erfolgen.

(2) [1]Der Träger der Einäscherungsanlage hat ein Einäscherungsverzeichnis zu führen, in das neben den Identitätsdaten des Verstorbenen der Tag der Einäscherung und der vorgesehene Bestattungsplatz einzutragen sind. [2]Das Verzeichnis mit der Eintragung ist 30 Jahre aufzubewahren. [3]Die Frist beginnt mit Ablauf des jeweiligen Sterbejahres.

(3) Die Benutzung der Einäscherungsanlage ist durch Satzung oder durch allgemeine Geschäftsbedingungen zu regeln.

§ 21
Bestatter, Totengräber

(1) [1]Wer gewerbs- oder berufsmäßig die Reinigung, Ankleidung oder Einsargung von Leichen vornimmt (Bestatter, Heimbürgen), oder wer die Tätigkeit

eines Totengräbers ausübt, darf nicht im Nahrungs-, Genußmittel- oder Gaststättengewerbe, als Hebamme oder Entbindungspfleger oder als Kosmetiker oder Friseur tätig sein oder dem Personenkreis im Sinne des § 33 IfSG angehören oder in diesen Bereichen von anderen beschäftigt werden. [2]Das Grundrecht der freien Berufsausübung (Artikel 12 Abs. 1 des Grundgesetzes, Artikel 28 Abs. 1 der Verfassung des Freistaates Sachsen) wird insoweit eingeschränkt.

(2) [1]Die Bestatter, Heimbürgen und Totengräber haben über das, was ihnen in ihrer Eigenschaft als Bestatter, Heimbürgen oder Totengräber anvertraut oder bekannt geworden ist, zu schweigen. [2]Sie sind zur Offenbarung befugt, wenn sie von der Schweigepflicht von dem gemäß § 10 Abs. 1 Verpflichteten entbunden wurden oder soweit die Offenbarung zum Schutz eines höherwertigen Rechtsgutes erforderlich ist.

(3) Die Tätigkeit der Bestatter und Totengräber unterliegt hinsichtlich der Einhaltung von Hygienevorschriften der Aufsicht des Gesundheitsamtes.

§ 22
Ausgrabung, Umbettung

(1) Während der gesetzlichen Mindestruhezeit darf die Totenruhe grundsätzlich nicht gestört werden.

(2) [1]Die Ausgrabung oder Umbettung einer Leiche bedarf einer schriftlichen Genehmigung des Gesundheitsamtes. [2]Die Ausgrabung oder Umbettung einer Urne bedarf der schriftlichen Genehmigung des Friedhofsträgers. [3]Dem Antrag auf Erteilung der Genehmigung zur Umbettung ist der Nachweis beizufügen, dass eine andere Grabstätte zur Verfügung steht.

(3) Für Ausgrabungen, die auf Grund anderer Rechtsvorschriften angeordnet oder zugelassen werden, gilt Absatz 1 nicht.

(4) Ausgrabungen oder Umbettungen dürfen in dem Zeitraum von 2 Wochen bis zu 6 Monaten nach dem Tode nicht zugelassen werden, sofern es sich nicht um Urnen handelt oder sofern die Ausgrabung oder Umbettung nicht richterlich angeordnet ist.

Vierter Abschnitt

Ordnungswidrigkeiten, Ermächtigungen und Schlussbestimmungen

§ 23
Ordnungswidrigkeiten

(1) Ordnungswidrig handelt, wer vorsätzlich oder fahrlässig

1. entgegen § 1 Abs. 3 einen Bestattungsplatz ohne Genehmigung anlegt, erweitert oder wiederbelegt,

2. entgegen § 3 Abs. 4 eine Leiche ohne Genehmigung auf einem privaten Bestattungsplatz bestattet oder bestatten lässt,

3. entgegen § 11 die Leichenschau nicht oder nicht unverzüglich veranlasst (§ 11 Abs. 1) oder die vorgeschriebene Benachrichtigung unterlässt (§ 11 Abs. 2),

4. entgegen § 12 Abs. 2 Satz 1 als Arzt die Leichenschau nicht oder nicht unverzüglich vornimmt,

5. entgegen § 13 Abs. 1 als Inhaber der tatsächlichen Gewalt dem Arzt oder von ihm hinzugezogenen Sachverständigen oder Gehilfen den Zugang verwehrt,

6. entgegen § 13 Abs. 2 oder § 14 Abs. 4 seiner Auskunftspflicht nicht nachkommt,

7. entgegen den §§ 12 bis 14 als Arzt die Todesbescheinigung (§ 14 Abs. 1) oder die vorläufige Todesbescheinigung (§ 12 Abs. 3) nicht, nicht unverzüglich oder nicht mit der gebotenen Sorgfalt ausstellt oder entgegen § 12 Abs. 4 keine Vertretung organisiert oder entgegen § 13 Abs. 4 die Polizei nicht benachrichtigt,

8. entgegen § 15 Abs. 1 eine nicht zulässige innere Leichenschau veranlasst oder durchführt,

9. entgegen § 15 Abs. 5 als obduzierender Arzt den Obduktionsschein nicht, nicht unverzüglich oder nicht mit der gebotenen Sorgfalt ausstellt,

10. entgegen § 13 Abs. 5 oder § 16 Abs. 4 das Gesundheitsamt nicht unverzüglich unterrichtet,

11. entgegen § 18 Abs. 1 in Verbindung mit § 19 als verantwortliche Person (§ 10) eine Leiche nicht, nicht rechtzeitig oder nicht ordnungsgemäß bestatten lässt oder wer eine Leiche beiseite schafft, um sie der Bestattung zu entziehen,

12. entgegen § 18a Abs. 2 oder 3 oder § 18b Abs. 2 oder 4 eine Leiche ohne Beurkundung des Sterbefalls durch das Standesamt, ohne die Unbedenklichkeitserklärung des Gesundheitsamtes oder ohne schriftliches Einverständnis der Staatsanwaltschaft oder des Ermittlungsrichters bestattet oder bestatten lässt,

13. entgegen § 18b Abs. 5 in Verbindung mit § 17 Abs. 3 eine Leiche zur Einäscherung in ein anderes Bundesland oder ins Ausland befördert, ohne zuvor eine zweite Leichenschau durch das Gesundheitsamt des Sterbeortes veranlasst zu haben,

14. entgegen § 18 Abs. 6 Satz 1 Fehlgeborene nicht, nicht rechtzeitig oder nicht ordnungsgemäß bestattet,

15. entgegen § 18 Abs. 7 der Beseitigungspflicht nicht, nicht rechtzeitig oder nicht ordnungsgemäß nachkommt,

16. entgegen § 19 Abs. 2 die Asche eines Verstorbenen nicht innerhalb von 6 Monaten nach der Einäscherung beisetzen lässt,

17. entgegen § 19 Abs. 3 in Verbindung mit § 16 Abs. 5 Satz 2 die zur Aufbewahrung von Leichen erforderliche Kühltemperatur nicht einhält oder die Beantragung der Bestattungsfristverlängerung versäumt,

18. entgegen § 21 Abs. 1 als Bestatter, Heimbürge oder Totengräber in einem nach § 21 Abs. 1 nicht zugelassenen Beruf oder Gewerbe tätig ist, wer einen Bestatter, einen Heimbürgen oder einen Totengräber in einem derartigen Beruf oder Gewerbe oder als Piercer, Tätowierer oder in ähnlichen Berufen beschäftigt oder wer gegen die in § 21 Abs. 2 Satz 1 vorgeschriebene Schweigepflicht verstößt oder

19. entgegen § 22 eine Leiche oder die Asche eines Verstorbenen ausgräbt oder umbettet.

(2) Ordnungswidrig handelt auch, wer vorsätzlich oder fahrlässig einer aufgrund von § 24 Abs. 1 erlassenen Rechtsverordnung zuwiderhandelt, soweit die Rechtsverordnung für einen bestimmten Tatbestand auf diese Bußgeldbestimmung verweist.

(3) Die Ordnungswidrigkeit kann mit einer Geldbuße, im Falle des Absatzes 1 Nr. 2 bis zu 5 000 EUR, geahndet werden.

(4) Zuständige Verwaltungsbehörde im Sinne des § 36 Abs. 1 Nr. 1 des Gesetzes über Ordnungswidrigkeiten ist

1. in den Fällen des Absatzes 1 Nr. 1 und 2 die Genehmigungsbehörde,

2. im Übrigen das jeweils zuständige Gesundheitsamt.

§ 24
Ermächtigungen

(1) Das Staatsministerium für Soziales wird ermächtigt, im Benehmen mit dem Staatsministerium des Innern, bei Regelungen nach Nummer 3 auch im Benehmen mit dem Staatsministerium der Justiz, durch Rechtsverordnung

1. Anforderungen an Bestattungsplätze (§ 1 Abs. 2), Leichenhallen (§ 2 Abs. 1) sowie an sonstige zur Aufbewahrung von Leichen dienende Räume (§ 16 Abs. 1) und an Bestattungseinrichtungen (§ 2 Abs. 1 Satz 2) festzulegen,

2. die im Rahmen des § 28 Abs. 1 IfSG notwendigen Schutzmaßnahmen bei hochinfektiösen Leichen zu treffen, insbesondere hinsichtlich a)der äußeren und inneren Leichenschau, b)des Umgangs mit der Leiche, c)ihrer Behandlung und Einsargung, d)der Anforderungen an die Aufbe-

wahrung der Leiche sowie der Beschaffenheit des Aufbewahrungsraumes, e)der Art und Weise des Leichentransportes, f)der Bestattungsart, g)der Untersagung bestimmter religiöser Bestattungsrituale,

3. die Bestimmungen über Inhalt, Form und Aufbewahrung der Todesbescheinigung (§ 14) und des Obduktionsscheines (§ 15 Abs. 5) zur Anpassung an neue Bedürfnisse der Praxis oder zur Vereinheitlichung der Verfahren im Bundesgebiet zu ändern und zu ergänzen, die Muster dieser Bescheinigungen entsprechend zu ändern sowie zu regeln, welchen sonstigen Stellen Todesbescheinigungen, Obduktionsscheine oder Kopien davon zu übermitteln sind oder übermittelt werden dürfen,

4. nähere Bestimmungen über die Beschaffenheit von Särgen und das Verfahren bei der Einsargung (§ 16 Abs. 3 und 4 sowie § 17 Abs. 5) zu treffen,

5. ergänzende Bestimmungen über die Beschaffenheit von Leichenfahrzeugen (§ 17 Abs. 1) zu treffen sowie zu bestimmen, welche Unterlagen bei der Beförderung von Leichen nach § 17 mitzuführen sind,

6. die Bestimmungen über Inhalt, Form und Aufbewahrung des Leichenpasses (§ 17 Abs. 3) zur Anpassung an neue Bedürfnisse der Praxis zu ändern und ergänzen, das Muster des Leichenpasses entsprechend zu ändern sowie zu bestimmen, welche Nachweise dem Antrag auf Ausstellung beizufügen sind,

7. das Verfahren für Bestattungen näher zu regeln und weitere Bestattungsarten mit anschließender Erdbestattung auf einem Friedhof in einem umweltgerecht abbaubaren Behältnis zuzulassen,

8. besondere Anforderungen an die Einäscherung (§ 20), insbesondere an die Beschaffenheit der Särge und der Urnen, festzulegen,

9. zu bestimmen, welche Angaben in den Einäscherungsverzeichnissen von den Trägern der Einäscherungsanlagen im Einzelnen zu machen (§ 20 Abs. 3) sind,

10. die Vorschriften der §§ 14 und 17 zur Anpassung an die für grenzüberschreitende Leichenbeförderungen im Raum der Europäischen Union und für die Beförderung aus dritten Ländern künftig geltenden rechtlichen Regelungen der Europäischen Union zu ändern und zu ergänzen.

(2) Vor dem Erlass von Rechtsverordnungen nach Absatz 1 Nr. 3 und 6 ist der Sächsische Datenschutzbeauftragte anzuhören.

(3) Die zur Durchführung dieses Gesetzes erforderlichen Verwaltungsvorschriften erlässt das Staatsministerium für Soziales.

§ 25
Sonderbestimmungen

Unberührt bleiben

1. internationale Vereinbarungen, insbesondere über die Leichenbeförderung,

2. Vorschriften über die Beförderung von Leichen auf Eisenbahnen, auf dem See- und auf dem Luftwege,

3. Vorschriften über den Umgang mit radioaktiven Leichen.

§ 26
Inkrafttreten

Dieses Gesetz tritt am Tage nach seiner Verkündung in Kraft.

Das vorstehende Gesetz wird hiermit ausgefertigt und ist zu verkünden.

Dresden, den 8. Juli 1994

Der Landtagspräsident
Erich Iltgen

Der Ministerpräsident
In Vertretung
Heinz Eggert
Der Staatsminister des Innern

Der Staatsminister für Soziales, Gesundheit und Familie
In Vertretung
Heinz Eggert
Der Staatsminister des Innern
