# Supplementary material for: The Stress-Strain Data of the Hip Capsule Ligaments Are Gender and Side Independent Suggesting a Smaller Contribution to Passive Stiffness
Source: PLoS One. 2016 Sep 29;11(9):e0163306. doi: 10.1371/journal.pone.0163306 (PMC5042535; doi:10.1371/journal.pone.0163306)
Supplement: S2 Table — Data display the mean, minimum and maximum values measured over all ten test cycles. F = female, M = male, L = left and R = right. (PDF) [file pone.0163306.s003.pdf]

Tissue Number	Age [years]	Gender	Side	Cause of Death	Iliofemoral (n=17)						Ischiofemoral (n=9)						Pubofemoral (n=17)					
					Strain [%]			Stress [N/mm²]			Strain [%]			Stress [N/mm²]			Strain [%]			Stress [N/mm²]		
Σ=17	83,65 ± 10,54	F:M (9:7)	L:R (13:8)		Mean	Minimum	Maximum	Mean	Minimum	Maximum	Mean	Minimum	Maximum	Mean	Minimum	Maximum	Mean	Minimum	Maximum	Mean	Minimum	Maximum
					129.79 ± 11.08	120.44 ± 8.04	132.15 ± 12.36	4.46 ± 1.93	3.01 ± 1.57	4.88 ± 2.15	128.67 ± 13.68	120.43 ± 9.46	130.18 ± 14.73	2.83 ± 1.89	2.14 ± 1.39	3.28 ± 2.02	133.24 ± 23.74	124.56 ± 20.36	135.34 ± 25.03	4.53 ± 2.79	3.08 ± 1.63	5.18 ± 3.78
1	95	F	L	Heart failure	131.34	122.26	133.07	4.77	3.25	5.04							117.64	110.33	119.21	11.58	3.42	16.44
2	78	M	L	Acute myocardinfarct	153.42	122.47	161.26	1.16	0.72	1.34							138.53	126.71	142.47	8.68	5.28	9.58
3	97	F	L	Acute myocardinfarct	129.83	124.22	130.84	3.19	2.69	3.30							115.00	111.57	115.98	2.92	2.58	3.00
4	85	F	R	Pneumonia	124.02	120.70	124.85	6.35	5.80	6.50	129.85	120.09	131.80	0.39	0.30	0.46	122.91	108.60	125.73	2.61	1.24	2.82
5	79	F	L	Pulmonary embolism	141.85	136.45	143.04	7.07	5.67	7.37							138.08	129.39	139.51	4.38	3.69	4.56
5	79	F	R	Pulmonary embolism	126.91	121.53	128.15	5.49	3.46	6.01							202.66	187.87	207.38	5.39	4.60	5.66
6	89	F	L	Pneumonia	132.61	126.89	134.24	5.49	4.60	5.73	154.92	126.71	159.84	6.06	3.82	6.55	116.69	110.04	117.53	1.01	0.60	1.07
7	68	M	L	Pulmonary embolism	113.77	106.90	115.75	2.44	0.44	3.04	145.27	141.99	146.04	3.86	3.65	3.92	132.72	127.93	134.66	2.26	2.05	2.42
7	68	M	R	Pulmonary embolism	127.11	123.74	128.35	4.77	3.90	4.97												
8	104	F	L	Heart failure	124.16	119.66	126.01	2.90	2.81	3.00												
9	67	F	L	Acute pancreatitis	125.23	114.66	127.53	2.00	1.23	2.13	115.37	113.93	115.78	0.73	0.72	0.74						
10	74	M	L	Heart-lung failure	144.85	135.09	148.15	3.03	2.33	3.32	119.21	111.17	120.14	3.22	2.10	3.36	123.82	121.34	124.38	4.30	3.60	4.53
10	74	M	R	Heart-lung failure							117.12	114.59	117.79	1.44	1.42	1.46	119.61	109.08	121.42	4.86	1.48	7.66
11	87	M	R	Heart failure	147.14	120.00	152.90	5.85	2.77	6.39	131.95	119.50	133.70	1.80	1.24	3.66	123.79	117.16	125.44	3.47	3.05	3.60
12	79	F	L	Cardiac arrest	118.68	116.20	119.30	2.60	2.54	2.70							117.00	113.55	117.59	3.60	3.01	3.69
12	79	F	R	Cardiac arrest													122.19	121.47	122.55	4.48	4.43	4.60
13	89	M	R	Cardiac arrest	126.27	111.09	128.43	4.44	1.45	5.48							181.63	158.89	187.67	8.40	6.50	8.88
14	84	M	L	Pneumonia	116.42	107.86	120.74	6.90	2.87	8.53							123.94	113.69	126.23	4.09	2.62	4.34
14	84	M	R	Pneumonia	122.83	117.75	124.06	7.41	4.59	8.10												
16	71	M	L	unknown							117.93	113.64	119.31	3.41	2.01	4.60	136.94	127.66	138.18	0.59	0.46	0.61
17	81	F	L	Heart failure							126.38	122.22	127.18	4.60	4.04	4.73	131.92	122.23	134.78	4.36	3.70	4.56
