# Supplementary material for: The Stress-Strain Data of the Hip Capsule Ligaments Are Gender and Side Independent Suggesting a Smaller Contribution to Passive Stiffness
Source: PLoS One. 2016 Sep 29;11(9):e0163306. doi: 10.1371/journal.pone.0163306 (PMC5042535; doi:10.1371/journal.pone.0163306)
Supplement: S3 Table — Mean values ± standard deviations for every ligament are presented in the caption. F = female, M = male, L = left and R = right. (PDF) [file pone.0163306.s004.pdf]

Tissue Number	Age [years]	Gender	Side	Cause of Death	Iliofemoral (n=17)		Ischiofemoral (n=9)		Pubofemoral (n=17)	
					Cross-section [mm²]	Elastic Modulus [N/mm²]	Cross-section [mm²]	Elastic Modulus [N/mm²]	Cross-section [mm²]	Elastic Modulus [N/mm²]
Σ=17	83,65 ± 10,54	F:M (9:7)	L :R (13:8)		53.47 ± 15.12	48.79 ± 21.43	19.23 ± 13.23	37.46 ± 20.42	15.20 ± 7.19	49.03 ± 32.07
1	95	F	L	Heart failure	49.85	49.28			11.62	148.15
2	78	M	L	Acute myocardinfarct	73.76	11.08			13.75	73.99
3	97	F	L	Acute myocardinfarct	68.02	31.53			12.95	46.07
4	85	F	R	Pneumonia	34.94	69.22	19.25	5.36	30.70	11.03
5	79	F	L	Pulmonary embolism	36.81	50.81			4.80	55.08
5	79	F	R	Pulmonary embolism	61.35	61.70			11.92	25.71
6	89	F	L	Pneumonia	41.67	37.48	12.27	45.98	17.43	26.08
7	68	M	L	Pulmonary embolism	70.21	58.06	13.55	41.14	14.56	28.26
7	68	M	R	Pulmonary embolism	51.40	69.40				
8	104	F	L	Heart failure	58.44	43.80				
9	67	F	L	Acute pancreatitis	74.38	22.99	18.72	22.09		
10	74	M	L	Heart-lung failure	60.14	25.73	15.51	47.24	19.41	75.62
10	74	M	R	Heart-lung failure			53.01	24.37	10.01	67.55
11	87	M	R	Heart failure	27.76	40.29	7.32	24.90	6.71	40.6
12	79	F	L	Cardiac arrest	43.13	35.32			13.36	49.45
12	79	F	R	Cardiac arrest					29.44	41.89
13	89	M	R	Cardiac arrest	40.37	51.49			8.40	52.53
14	84	M	L	Pneumonia	72.94	75.63			16.11	45.12
14	84	M	R	Pneumonia	43.94	95.67				
16	71	M	L	unknown			14.59	74.22	23.52	7.62
17	81	F	L	Heart failure			18.85	51.84	13.71	38.73
